# Immunization with a multi-antigen targeted DNA vaccine eliminates chemoresistant pancreatic cancer by disrupting tumor-stromal cell crosstalk

**DOI:** 10.1186/s12967-023-04519-3

**Published:** 2023-10-09

**Authors:** Hongquan Qin, Jiali Chen, Katia Bouchekioua-Bouzaghou, Ya-Ming Meng, Jordi Bach Griera, Xue Jiang, Xiangzhan Kong, Minghui Wang, Qiuping Xu, Ping-Pui Wong

**Affiliations:** 1 Guangdong Provincial Key Laboratory of Malignant Tumor Epigenetics and Gene Regulation, Guangdong-Hong Kong Joint Laboratory for RNA Medicine, Sun Yat-sen Memorial Hospital, Sun Yat-sen University, Guangzhou, 510120 China; 2 Medical Research Center, Sun Yat-sen Memorial Hospital, Sun Yat-sen University, Guangzhou, 510120 China; 3 Department of Obstetrics and Gynecology, Center for Reproductive Medicine; Guangdong Provincial Key Laboratory of Major Obstetric Diseases; Guangdong Provincial Clinical Reserach Center for Obstetrics and Gynecology; Guangdong-HongKong-Macao Greater Bay Area Higher Education Joint Laboratory of Maternal-Fetal Medicine, The Third Affiliated Hospital of Guangzhou Medical University, Guangzhou, 510150 China; 4 Key Laboratory for Reproductive Medicine of Guangdong Province, The Third Affiliated Hospital of Guangzhou Medical University, Guangzhou, China; 5 Department of Thoracic Surgery, Sun Yat-sen Memorial Hospital, Sun Yat-sen University, Guangzhou, 510120 China

**Keywords:** Chemoresistance, Immunotherapy, Tumor microenvironment, Cytokines

## Abstract

**Background:**

Pancreatic ductal adenocarcinoma (PDAC) is characterised by limited responses to chemoimmunotherapy attributed to highly desmoplastic tumor microenvironment. Disrupting the tumor-stromal cell crosstalk is considered as an improved PDAC treatment strategy, whereas little progress has been made due to poor understanding of its underlying mechanism. Here, we examined the cellular role of melanoma associated antigen A isoforms (MAGEA) in regulating tumor-stromal crosstalk mediated chemoresistance.

**Methods:**

We used clinical samples to explore the correlation between MAGEA expression and patient prognosis in multiple cancers. We utilized cancer cell lines, patient derived organoids and orthotopic PDAC model to examine the function of MAGEA in chemoresistance. We performed biochemical, proteome profiler array and transcriptional analysis to uncover a mechanism that governs tumor-stromal crosstalk. We developed a multi-MAGEA antigen targeted DNA vaccine and tested its effect on PDAC tumor growth.

**Results:**

We establish MAGEA as a regulator of the tumor-stromal crosstalk in PDAC. We provide strong clinical evidence indicating that high MAGEA expression, including MAGEA2, MAGEA3 and MAGEA10, correlates with worse chemotherapeutic response and poor prognosis in multiple cancers, while their expression is up-regulated in chemoresistant PDAC patient derived organoids and cancer cell lines. Mechanistically, MAGEA2 prohibits gemcitabine-induced JNK-c-Jun-p53 mediated cancer cell apoptosis, while gemcitabine stimulated pancreatic stellate cells secretes GDF15 to further enhance the gemcitabine resistance of MAGEA2 expressing cells by activating GFRAL-RET mediated Akt and ERK1/2 dependent survival pathway. Strikingly, immunization with a DNA vaccine that targeting multiple MAGEA antigens, including MAGEA2, MAGEA3 and MAGEA10, elicits robust immune responses against the growth of gemcitabine resistant tumors.

**Conclusions:**

These findings suggest that targeting MAGEA-mediated paracrine regulation of chemoresistance by immunotherapy can be an improved pancreatic cancer treatment strategy.

**Supplementary Information:**

The online version contains supplementary material available at 10.1186/s12967-023-04519-3.

## Background

Pancreatic ductal adenocarcinoma (PDAC) is one of the deadliest cancers worldwide, while there is no effective single/combined chemotherapy and immunotherapy treatment strategy available for PDAC patients, primarily because of its desmoplastic and immunosuppressive tumor microenvironment [1–5]. Poor understanding of the mechanistic role of tumor-stromal cell interaction in chemoresistance has significantly hindered the development of such combination treatment strategies in pancreatic cancer.

Gemcitabine (Gem) and fluorouracil (5′FU) are standard chemotherapies which are often used in combination therapies for a wide range of cancers, especially for PDAC [6–8]. Unfortunately, only a small number of Gem or 5′FU treated patients show good clinical outcome from these chemotherapies, and most develop resistance. Recent studies have suggested that stromal cells, like pancreatic stellate cells (PSCs), may involve in chemoresistance and cancer progression [5, 9, 10]. For example, PSC-secreted SDF-1 stimulates Gem resistance and cell invasion in pancreatic cancer by activating its receptor CXCR4, while PSC-derived TGF-β affects tumor cell properties including survival, metastasis, angiogenesis and chemoresistance [11, 12]. Importantly, growth/differentiation factor 15 (GDF15) has been shown to associate with PDAC progression [13], while it can bind to its newly identified receptor GDNF family receptor α-like (GFRAL) to activate the recruitment of its co-receptor, a receptor tyrosine kinase named as RET (Rearranged during transfection), in order to modulate energy homeostasis [14]. However, the regulatory role of GDF15-GFRAL-RET signalling axis in cancer chemoresistance is unexplored.

The MAGE (melanoma associated antigen) protein family is a group of proteins that are well-conserved and possess a common MAGE homology domain [15]. These proteins can be categorized into two types based on their expression patterns. Type I MAGEs, such as MAGEA, MAGEB, and MAGEC, are associated with cancer, while type II MAGEs are expressed in various tissues [15–17]. Notably, MAGEA family members have been shown to exhibit strong immunogenicity and are recognized as promising targets for cancer immunotherapy [18–20]. Indeed, peptides derived from MAGEA proteins have been demonstrated to be presented by HLA (human leukocyte antigen) molecules to activate T cells, and cancer patients have been observed to possess anti-tumor CD8^+^ T cells that recognize MAGEA antigens [21]. Moreover, previous studies have shown that treatment with recombinant MAGEA3 peptide combined with an adjuvant can stimulate the production of antibodies and activate CD4^+^ and CD8^+^ T cell responses [22]. Apart from their immune functions, MAGEA2, MAGEA3, and MAGEA10 are well-known for their cellular roles in regulating cell proliferation, migration, and apoptosis, while sharing high structural similarity and potentially having overlapped cellular functions [23–25]. Accumulating evidence supports their critical roles in cancer progression. For instance, MAGEA3 overexpression has been shown to increase tumor growth and metastasis, while silencing MAGEA3 inhibits cancer cell proliferation [26]. MAGEA3 expression has also been implicated in inhibiting autophagy through the formation of a complex with E3 ubiquitin ligase (TRIM28) to target AMPK degradation during tumorigenesis [25, 27]. Importantly, our research and that of others have shown that MAGEA2 and MAGEA3 can cooperate to inhibit p53-dependent apoptosis, while enhancing the cell proliferation pathway mediated by estrogen receptor-α in tamoxifen-resistant breast cancer [24, 28]. Additionally, MAGEA10 has been shown to be involved in regulating cell–cell interaction and adhesion [23]. Nevertheless, their precise functions in cancer chemoresistance and progression remain unclear.

Here, we exploit the cellular role of MAGEA in tumor-stromal mediated chemoresistance and cancer progression, and test whether the use of our newly developed multi-MAGEA antigen targeted DNA vaccine can tackle cancer chemoresistance.

## Methods

### Clinical specimens and data analysis

Human PDAC specimens were collected from Sun Yat-sen Memorial hospital, with fully signed informed consent forms and approval obtained from the patients and the ethical committee of our hospital (Approval no.: SYSEC-KY_KS-2018-127) respectively. These patients received adjuvant Gem-based treatment strategy and their therapeutic outcome and clinicopathological data were evaluated and recorded. For the Kaplan–Meier (KM) plotter database analysis, it was done as described before (http://kmplot.com/analysis/) [29]. For TCGA dataset analysis, the cancer patients included in this study must meet the criteria: (1) patients with complete clinical information (expression and clinicopathological data); (2) patients documented with complete clinical outcome data including survival, lymph node metastasis, tumor size, and cancer stages. For the TIMER2.0 analysis, the TIMER2.0 Gene Outcome module was utilized to examine TCGA cancer survival data, employing a Cox proportional hazards model, and to assess the impact of MAGEA2, MAGEA3 or MAGEA10 expression according to the web tool developer's instruction [30].

### Immunohistochemistry experiment

Paraffin embedded tissue sections were first subjected to antigen retrieval procedure, which were boiled in the presence of 10 mM citrate buffer (pH 6). Soon after, the sections were rinsed with PBS and blocked in 1% normal goat serum (NSG) together with 0.1% TritonX-100 (TX-100) in PBS for 1 h. The sections were then immunostained for either pan-MAGEA ((6C1): Santa Cruz Biotechnology-sc-20034), GFRAL (Abcam-ab107719), GDF15 (Santa Cruz Biotechnology-sc-377195), CD4 (D7D2Z, Cell signaling-#25229), CD8 (D4W2Z, Cell signaling-#98941) or Granzyme B (D6E9W,Cell signaling-# 46890S) antibody at 4 °C for 16 h. Afterwards, they were then washed with PBS three times, and diluted biotinylated secondary antibody (polyclonal anti-mouse biotinylated, Dako) was then added to the sections for 45 min. At the end, the sections were incubated with enzymatic Avidin–Biotin Complex (ABC)-3,3'-diaminobenzidine (DAB) staining (Vector Laboratories) and counterstained with hematoxylin. Each section was assigned a score 0, 1, 2 or 3 for the area and intensity of staining respectively (i.e. The final staining score of each slide = the score of the area × the score of the intensity). The median of staining score was calculated and set as a cutoff threshold for stratifying patients into high and low MAGEA expression groups. In the case of DT6066 orthotopic and KPC spontaneous tumors, the tumor sections were obtained from our previous publication and the IHC data analysis was carried out as described before [31]. For the cancer progression study, the tumor IHC staining of pan-MAGEA was measured in PDAC patients who developed recurrence less than 2-year period post gem-based treatment.

### Tissue culture

MIA PaCa-2, CFPAC-1, T3M4 and Capan-1 cells were purchased from ATCC and were cultured in DMEM/RPMI1640 plus 10% fetal bovine serum (FBS) and 1% penicillin/streptomycin inside a CO_2_ incubator at 37 °C. These cell lines were allocated into gemcitabine sensitive (GemS) or resistant (GemR) groups based on their IC50 doses and previous findings [32]. DT6066 and TB32048 pancreatic cell lines were kindly given by Professor Kairbaan Hodivala-Dilke [3, 31]. Human PSCs were generated as previously described [33]. For Gem or 5′FU-resistant mouse/human pancreatic cell line generation, cell lines were cultured with increasing doses of gemcitabine or 5′FU for 6 months until the cells were completely resistant to gemcitabine (2000 nM) or 5′FU treatment (4000 nM) which was verified by using CCK8 cell viability assays as described below. Human PDAC patient derived tumor organoids were generated as done previously [34].

### Western blot analysis

Cancer cells/PSCs were first harvested and lysed by incubating with NP40 lysis buffer (Invitrogen) supplemented with protease and phosphatase inhibitor cocktails on ice for up to 1 h. Afterwards, the Bio-Rad Dc Protein Assay Kit (Bio-Rad Laboratories) was used to measure the protein concentration in each sample, while 15–30 μg of the protein from each sample was loaded onto 8–12% polyacrylamide gels. The protein was transferred onto a nitrocellulose membrane and blocked with 5% milk in PBS-T (i.e. PBS with 0.1% Tween-20) for 1 h, which were then subjected to an overnight incubation of primary antibody (i.e. 1:1000 dilution) in 2% milk in PBS-T at 4 °C. The blots were then washed with PBS-T and incubated with 2% milk PBS-T containing relevant horseradish peroxidase (HRP)-conjugated antibody (diluted 1:1000) for 1 h. The following antibodies were employed in this study: mouse monoclonal anti-MAGEA3 (Proteintech-60054-1), rabbit polyclonal anti-MAGEA2 (Proteintech-144881-1-AP), rabbit polyclonal anti-MAGEA10 (Sigma Aldrich-HPA003333), rabbit polyclonal anti-SPAK/JNK antibody (Cell signaling-#9252), mouse monoclonal anti-phospho-SPAK/JNK antibody (Thr183/Try185, Cell signaling-#9255), rabbit polyclonal anti-c-Jun antibody (Cell signaling-#9165), rabbit polyclonal anti-phospho-c-Jun antibody (Ser 63, Cell signaling-#91952), mouse monoclonal anti-p53 antibody (Santa Cruz-sc-126), Rabbit polyclonal anti-phospho-p53 (Ser 46, Cell signaling-#2521), rabbit monoclonal anti-Akt (Cell Signaling-#4691), rabbit monoclonal anti-phospho-Akt (Ser 473, Cell Signaling-#4060), rabbit polyclonal anti-phospho-RET (Tyr 905, Cell signaling-#3221), rabbit monoclonal anti-RET ((E1N8X) XP^®^, Cell signaling-#14556), rabbit polyclonal anti-GFRAL (Abcam-ab107719), mouse monoclonal anti-GDF15 (Santa Cruz Biotechnology-sc-377195), rabbit polyclonal anti-phospho-ERK1/2 (Thr 202/Tyr204, Cell signaling-#9101), rabbit monoclonal anti-ERK1/2 (Cell signaling-#4695), mouse monoclonal anti-Hsc70 (Santa Cruz Biotechnology-sc7298).

### Cell viability measurement

CCK8 proliferation assays were done as suggested by the manufacturer’ (Jiangsu KeyGENBioTECH Corp., Ltd). Briefly, 1000–3000 cells were plated onto a 96 well plate, which were then incubated with a titrated concentration of Gem or 5′FU for 24 h. 20 μL of CCK8 proliferation assay was added to each well and left for an hour at 37 °C. The absorbance at 490 nm for each well was recorded using a 96-well plate reader. For pharmacological activation of JNK, MIA PaCa-2/CFPAC-1 derived MAGEA2 or VA clones were treated with placebo, anisomycin (370 nM) (R&D, Cat no. 1290/10), Gem (the IC50 doses of MIA PaCa-2/CFPAC-1 VA clone) or Gem plus anisomycin for 48 h. For conditioned medium (CM) experiments, PSCs were cultured with phenol red free and serum free medium −/+ Gem (the IC50 dose of PSC = 5 nM) for 16 h, and then starved with fresh phenol red free and serum free medium for another 72 h. The conditioned medium was supplemented with placebo/Gem, and then applied to serum starved PDAC cells and the cell viability measured by CCK8 proliferation assay every 24 h for up to 120 h. For the trypsin treatment experiment, the conditioned medium (CM), with or without beads with immobilized trypsin (TPCK treated agarose Resin, Thermofisher), was incubated overnight on a roller at 37 °C. The beads were removed from the CM by performing centrifugation at 1000*g* 10 min. The collected supernatant was then added with placebo/Gem and finally used for tumor cell viability test.

### Plasmids, siRNA and transfection conditions

PDAC cells were transfected with pcDNA3.1 (+) MAGEA2, MAGEA3 or MAGEA10 overexpression plasmids (purchased from IGE Biotechnology) using a jetPRIME^®^ transfection reagent (Polyplus-transfection) following the manufacturer’s instruction. Briefly, stable clones were cultured in medium containing 800–1000 μg/mL G418 (Gibco Geneticin), which were then subjected to western blot and RT-PCR analysis. The RT-PCR primer sequences were given in Additional file 1: Table S1. For siRNA transfection, sub-confluent PDAC cells were transiently transfected using Lipofectamine RNAiMAX (Invitrogen) with 50 nM of either non-silencing control siRNA or siRNAs targeting MAGEA2, MAGEA3, and MAGEA10 (purchased from Genepharma), either transfected alone or in combination as indicated in the figure. After transfection, cells were treated with or without Gemcitabine (Gem) and subjected to western blotting/RT-PCR analysis as well as CCK8 survival assays. For human PSCs, the cells were transfected with 50 nM siNSC or GDF15 targeting siRNA (purchased from Genepharma), and the transfected PSCs were treated with Gem.

### Proteome profiler array analysis

PDAC cells were grown in either normal culturing medium −/+ Gem or conditioned medium of Gem-stimulated PSCs. After 48-h incubation, the whole-cell lysate sample was then harvested and subjected to phospho-receptor tyrosine kinase (Proteome-Profilers ARY001B, R&D systems) and apoptosis array analysis (Human apoptosis antibody array membrane ab134001, Abcam) respectively. Image J software was used to quantify the array results. For cytokine array, PSCs were grown in culturing medium with placebo or Gem for 16 h, and then incubated with serum free medium for another 72 h. The protein samples were extracted and used for human XL cytokine arrays (Proteome-Profiler ARY022, R&D systems).

### FACS analysis

Splenocytes were first stained with PE anti-mouse CD8 antibody (Biolegend-#100707) and APC/Fire™ 750 anti-mouse CD4 antibody (Biolegend-#100459). For intracellular staining, splenocytes were then fixed and permeabilized with IntraPrep Permeabilization Reagent (Beckman, A07803) with APC-conjugated anti-mouse IFN-γ (Biolegend-#505810). The cells were then used for FACS analysis using cytoflex S (Beckham coulter) and the data was processed using Flowjo software. For the apoptosis assays, MAGEA2-expressing MIA PaCa-2 and VA cells treated with or without Gem were harvested and subjected to FACS analysis using a AF647 Annexin V apoptosis detection kit with PI (GOONIE -# 100-102-100). The proportions of viable cells and apoptotic cells in the total cell population were evaluated. For the analysis of FasL expression, MAGEA2-expressing MIA PaCa-2 and VA cells treated with or without Gem were incubated with PE conjugated anti-human Fas-L (BioLegend-#306406) and then subjected to FACS analysis.

### Spheroid growth and invasion assay

PDAC cell lines were first suspended in normal culturing medium with a final concentration of 200 cells per 200 μL. The resuspended cells were then seeded onto ultra-low attachment 96-well round-bottomed plates (Corning Inc.), which were then left untouched for 4 days. On day 4, 100 μL of the culturing medium from each well was carefully removed and replaced with 100 μL of 30% Matrigel (BD Bioscience) in normal medium, with or without Gem. Spheroid growth was monitored and photographed at 10 × magnification using an inverted Axiovert microscope (Zeiss) every day for up to 5 days, and the spheroid diameters were measured using ImageJ software. For conditioned medium experiments, 100 μL of the culturing medium was replaced with 30% Matrigel in 100 μL serum free conditioned medium derived from placebo or Gem-treated PSCs, together with or without Gem on day 4 instead. For invasion measurement with DT6066/TB32048 derived parental control and GemR spheroids, the relative invasion was determined from images of each spheroid using ImageJ by subtracting the solid spheroid area from the maximal invaded spheroid area and normalizing to the control.

### Orthotopic PDAC tumor model

1 × 10^6^ MIA PaCa-2 derived VA or MAGEA2 (C10) cells were mixed with 30 μL Matrigel /PBS (1:1) and implanted into the pancreases of 40 female nude mice. Tumor burden was measured by performing MRI once a week as previously described [3]. When the mouse tumor sizes reached around 200 mm^3^, the tumor bearing mice were randomly allocated into different treatment groups, which were treated with 50 mg/kg Gem or placebo once a week via an intraperitoneal injection for up to 4 weeks. Once the treatment finished, the mice were sacrificed, tumors, lungs and inguinal lymph nodes excised, which were then either fixed in 4% formaldehyde for 24 h or snap frozen for further histopathological analysis.

### Immunization with multi-MAGEA antigen targeted DNA vaccine

The multi-antigen targeted DNA plasmid and animal immunization process was designed and performed as described previously [35, 36]. Briefly, C57BL/6 mice were subcutaneously implanted with 3 × 10^6^ GemR or WT DT6066 cells. Once the tumors reached similar sizes, the mice were randomly allocated into two groups which were then immunized with either 50 μg pCDNA 3.1(+) empty vector or pCDNA 3.1(+) MAGEA2-MAGEA3-MAGEA10 vector (full length murine cDNAs encoding each MAGEA family member linked by T2A spacers [37]) together with 50 μg poly (I:C) (InvivoGen) once a week for up to 3 times. After the treatment, the experimental animals were culled, while the tumors/organs were harvested and fixed for further histopathological studies.

To test the immunogenic effect of our DNA vaccine in vitro, the subclass of the major histocompatibility complex (MHC-I) expression on GemR DT6066 cell line was first identified by performing FACS analysis with H-2Kb (MHC class I (H-2kb) monoclonal antibody (AF6-88.5.5.3), PE, Thermofisher Scientific-#12-5958-82) or H-2Db (MHC class I (H-2Db) monoclonal antibody (28-14-8), APC, Thermofisher Scientific-#17-5999-82) antibody. On confirming its MHC-I subclass as H-2Db, the T cell epitope prediction of mouse antigen MAGEA2/MAGEA3/MAGEA10 was performed by using Immune Epitope Database (IEDB). The information about peptide libraries for in vitro simulation of MAGEA2/MAGEA3/MAGEA10 and myelin oligodendrocyte glycoprotein (MOG)-encoded epitopes was provided in Additional file 2: Table S2. All these peptides were synthetized by IGE Biotechnology (China) and diluted in CAN (acetonitrile) plus H_2_O (1:3) at 1 mg/mL and stored at − 80 °C.

For IFN-γ ELISpot assays, the immunized C57BL/6 mice were culled, and their spleens were harvested and filtered through a 40 μm cell strainer to remove debris. The erythrocytes were lysed with RBC lysis buffer (Biolegend), and the isolated cells were rinsed and then cultured in RPMI1640 containing 10% FBS (ThermoFisher scientific). 5 × 10^5^ splenocytes were seeded per well and stimulated with 10 μg/mL epitopes specific for either MAGEA2, MAGEA3, MAGEA10 or negative control peptide for 36 h in ELISpot white bottom multiwall plates (3321-4HPW-2, Mabtech). Positive control was done by incubating the cells with 20 ng/mL phorbol myristate acetate (PMA, Sigma-Aldrich) plus 1 μg/mL ionomycin (Sigma-Aldrich). After 36 h, IFN-γ-producing cells were detected by IFN-γ ELISpot kit (Mabtech) and quantified by using an ELISpot Analyser (AID). All animal procedures were approved by the Institutional Animal Care and Use Committee (IACUC) of Sun Yat-sen University and followed the ARRIVE guidelines.

### General statistics

The information about statistical details in this manuscript is provided in the figure legends. The measurements of all statistical values were carried out using Graphpad Prism. Error bars in the experiments indicate standard error of the mean (SEM) for a minimum of three experimental repeats. Some of the schematic figures are obtained from the Servier Medical Art. Servier Medical Art by Servier is licensed under a Creative Commons Attribution 3.0 Unported License (https://creativecommons.org/licenses/by/3.0/). All authors had access to the study data and reviewed and approved the final manuscript.

## Results

### MAGEA expression determines patient prognosis and chemotherapeutic responses in different cancers

**Fig. 1:**
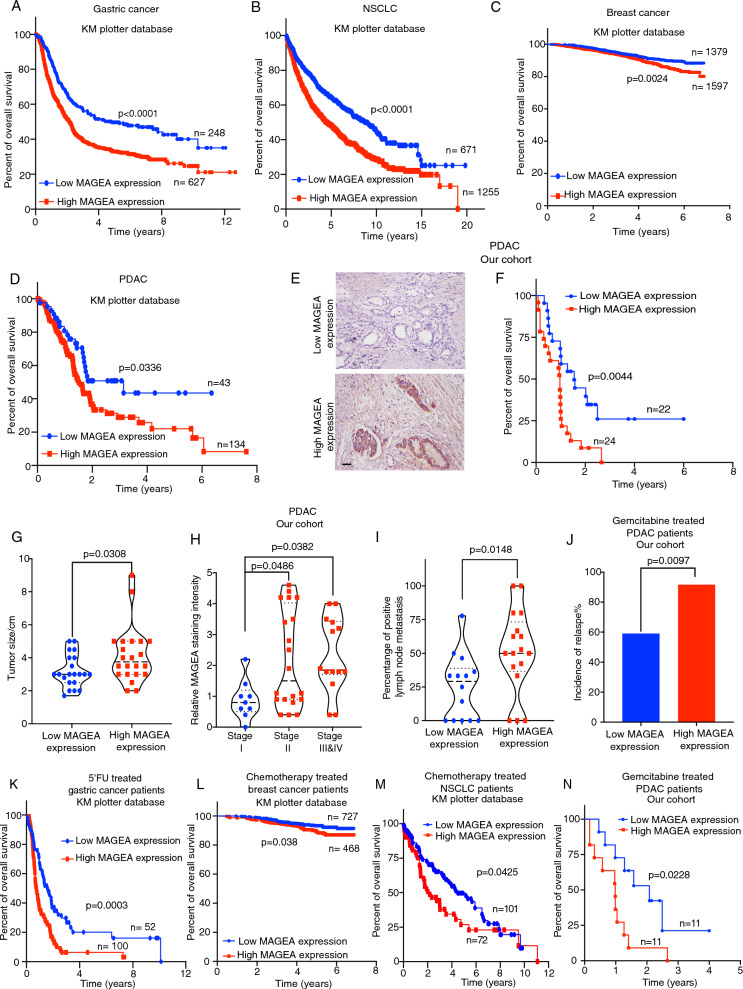
MAGEA expression level strongly associates with therapeutic response and patient prognosis in multiple cancers. Analysis of MAGEA expression in human gastric cancer (n = 152–875 patient samples, KM plotter database), non-small cell lung carcinoma (NSCLC) (n = 1926 patient samples, KM plotter database), breast cancer (n = 2976 patient samples, KM plotter database), pancreatic ductal adenocarcinoma (PDAC) (n = 177 patients, KM plotter database, n = 22–46 patients, our cohort), and on the basis of KM plotter database and our collected patient cohort. **A**–**F** Kaplan–Meier survival study of the MAGEA expression in human GC, NSCLC, BC and PDAC patients. Representative images of MAGEA IHC staining from tumor sections are given here. **G**–**I** Correlation studies between MAGEA expression and tumor size/enhanced cancer progression/lymph node metastases in PDAC patients. Means ± S.E.M are given. **J**–**N** The chemotherapy treated cancer patients with high MAGEA expression were associated with increased incidence of relapse or/and worse overall survival in gastric cancer (n = 152 patients, KM plotter database), breast cancer (n = 1195 patients, KM plotter database), NSCLC (n = 173 patients, KM plotter database) and PDAC patients (n = 22 patients, our cohort) respectively. **A**–**D**, **F**, **K**–**N** Log-rank (Mantel-Cox) test. **G**, **I** Student’s *t* test. **H** One-way ANOVA. **J** Chi-square test. Scale bar in (**E**) represents 100 μm

Given the high association of type 1 MAGE expression with tumor tissues [15], we focused our investigation on the role of individual family members in determining patient prognosis. The type 1 MAGEA family comprises three major subclasses (MAGEA, MAGEB, MAGEC) with numerous members [15]. To identify potential correlations with patient survival, we utilized the online KM plotter database tool. Our analysis revealed that only the expression of MAGEA2, MAGEA3, or MAGEA10, among the MAGEA family members, was significantly correlated with poor survival in PDAC patients (Additional file 3: Fig. S1A–K). In contrast, the expression of MAGEB, and MAGEC family members was not associated with patient survival (Additional file 3: Fig. S1L–X), suggesting that these three MAGEA family members may have a specific and distinct role in cancer progression compared to others. To further validate the clinical significance of our findings, we conducted a pan-MAGEA analysis using the KM plotter and TIMER2.0 databases, which includes data from various cancers, summing the expression of MAGEA2, MAGEA3, and MAGEA10. Additionally, we performed immunohistochemistry (IHC) examination on our PDAC patient cohorts using an antibody against the entire MAGEA family, as specific IHC antibodies against individual MAGEA2, MAGEA3, or MAGEA10 were not available, to corroborate our observations. Strikingly, our clinical studies indicated that high expression of MAGEA correlated with poor overall survival for gastric cancer (GC) (Fig. 1A), non-small cell lung cancer (NSCLC) (Fig. 1B), breast cancer (BC) (Fig. 1C) and PDAC (Fig. 1D–F, Additional file 3: Fig. S1Y). Moreover, we showed that the patients with high MAGEA expression were stratified into those with increased tumor size (Fig. 1G), enhanced cancer progression (Fig. 1H) elevated lymph node metastasis (Fig. 1I), increased incidence of relapse (Fig. 1J), and worse overall survival in chemotherapy treated GC, BC, NSCLC and PDAC cancer patients (Fig. 1K–N). Our clinical studies have revealed that the expression of MAGEA, particularly MAGEA2, MAGEA3, and MAGEA10, but not other family members, may play a crucial role in determining tumor growth, chemoresistance, and cancer progression in multiple cancers.

### MAGEA expression regulates gemcitabine resistance and tumorigenicity

**Fig. 2:**
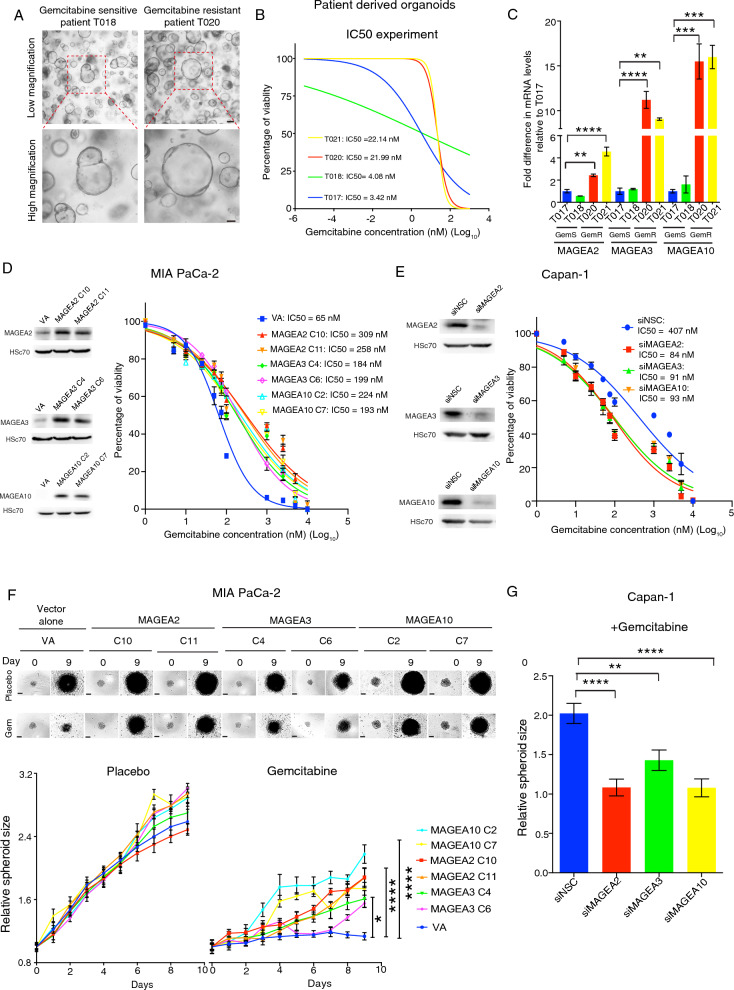
MAGEA overexpression is functionally linked to gemcitabine resistance. **A** Representative bright field images of GemS and GemR patient derived tumor organoids. **B** Gem IC50 dose examination of the tumor organoids from GemS (Patient T017 and T018) and GemR PDAC patients (Patient T020 and T021) respectively. **C** Analysis of MAGEA mRNA expression in GemS and GemR PDAC patient derived organoids (n = 3 experimental repeats). **D** Western blot analysis of MIA PaCa-2-stable clones expressing MAGEA2 (C10 and C11), MAGEA3 (C4 and C6) or MAGEA10 (C2 and C7) plus an empty vector transfected (VA) clone. Line graph shows the Gem IC50 experiment of MAGEA2/MAGEA3/MAGEA10- or VA-expressing cells (n = 9 experimental repeats). **E** Western blot analysis of Capan-1 cells transiently transfected with non-silencing control (siNSC) or MAGEA2, MAGEA3 or MAGEA10 targeting siRNA. Line graph shows the Gem IC50 experiment of Capan-1 cells transfected with siNSC or MAGEA2/MAGEA3/MAGEA10 targeting siRNA (n = 6 experimental repeats). **F** Spheroid assay of stable MAGEA2/MAGEA3/MAGEA10-expressing MIA PaCa-2 cells in the presence/absence of Gem treatment (n = 4–6 experimental repeats). **G** Bar chart represents the relative spheroid size of siMAGEA2/MAGEA3/MAGEA10 transfected Capan-1 cells after treated with Gem for 9 days. Means ± S.E.M. (n = 9 experimental repeats). *p < 0.05; **p < 0.01; ***p < 0.001; ****p < 0.0001. **C**, **G** One-way ANOVA. **F** Two-way ANOVA. Scale bars in (**A**) 200 μm (upper panel), 100 μm (lower panel), (**F**) represent 200 μm

To investigate whether MAGEA antigens regulated cancer chemoresistance and tumorigenicity, we first generated tumor organoids from gemcitabine-sensitive (GemS Patient T017 and T018) or -resistant PDAC patients (GemR Patient T020 and T021) respectively (Fig. 2A) and confirmed their sensitivity to Gem in vitro by performing IC50 experiments (Fig. 2B). Importantly, our data showed that GemR patient derived organoids expressed higher levels of MAGEA2, MAGEA3 and MAGEA10 as compared with the organoids derived from GemS patients (Fig. 2C). We next tested if overexpression of these MAGEA family members could determine Gem resistance. GemS MIA PaCa-2 and CFPAC-1 cells (whose sensitivity to Gem was confirmed in a previous publication [32]) were initially transfected with either MAGEA2/MAGEA3/MAGEA10 overexpression vector or empty vector (VA), while several individual clones were expanded and their overexpression was confirmed by the western blot analysis (Fig. 2D, Additional file 4: Fig. S2A). IC50 experiment indicated that stably MAGEA2-, MAGEA3- or MAGEA10-expressing clones were more resistant to Gem compared to the VA clone (Fig. 2D, Additional file 4: Fig. S 2A). Interestingly, CCK8 proliferation assays showed that MAGEA2/MAGEA3/MAGEA10 overexpression did not possess an inherent proliferation advantage of PDAC cell lines under normal culturing medium (Additional file 4: Fig. S2B). We next transiently transfected GemR PDAC cell lines (as tested in a previous publication [32]), Capan-1 and T3M4, with non-silencing control (siNSC) or MAGEA2/MAGEA3/MAGEA10 targeting siRNA, and then monitored the change in Gem IC50 dose in these transfected cells. Strikingly, silencing MAGEA2, MAGEA3 or MAGEA10 in Capan-1 and T3M4 cells rescued their sensitivity to Gem, when compared with siNSC transfected cells (Fig. 2E, Additional file 4: Fig. S2C). Moreover, spheroid assay results showed that the tumor spheroids stably expressing MAGEA2/MAGEA3/MAGEA10, but not the empty vector transfected cells derived organoids, were capable of growing in the presence of Gem (Fig. 2F), whereas silencing MAGEA2/MAGEA3/MAGEA10 inhibited the growth of Capan-1 and T3M4 derived spheroids under Gem treatment (Fig. 2G, Additional file 4: Fig. S2D). Building on previous research that has demonstrated the collaborative effects of MAGEA family members in cellular processes and signalling pathways [24, 38], we investigated whether this phenomenon was also observed in our study. Notably, our results indicated that when MAGEA2, MAGEA3, and MAGEA10 were silenced simultaneously using siRNAs, the survival of Capan-1 and T3M4 cells in the presence of Gem was significantly reduced compared to cells transfected with siRNAs targeting one or two of the family members (Additional file 4: Fig. S2E-H). These results highlight the potential therapeutic significance of targeting all three MAGEA family members simultaneously in the management of PDAC chemoresistance.

### MAGEA2 protein inhibits gemcitabine-induced apoptosis pathway via repression of the JNK-c-Jun-p53 signalling axis

**Fig. 3:**
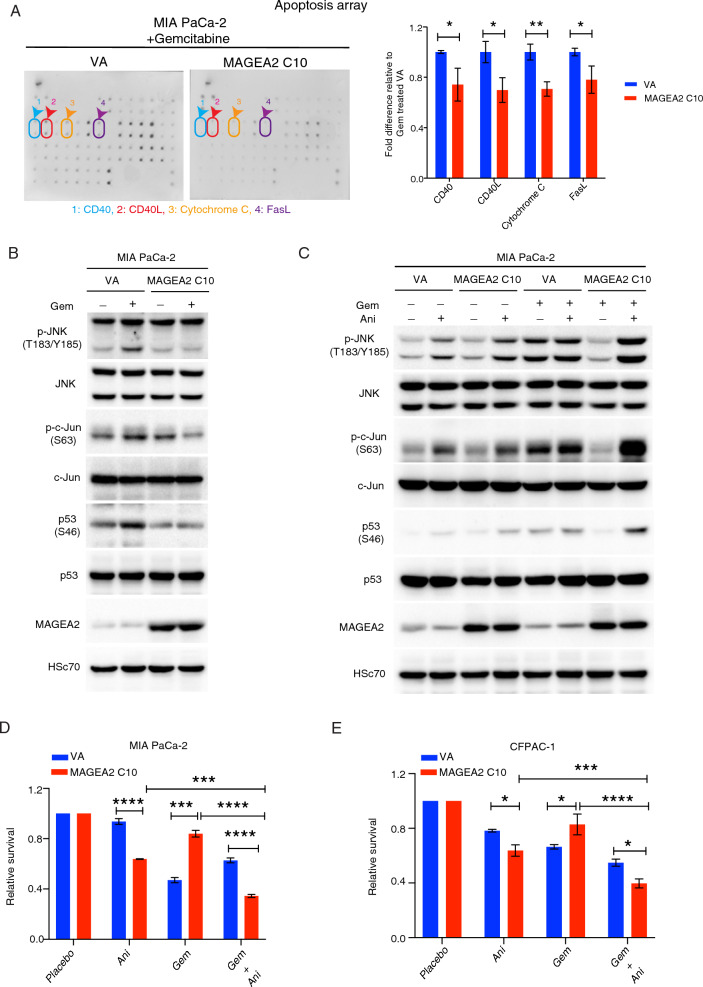
MAGEA2 overexpression represses gemcitabine-induced apoptotic pathways via inhibition of the JNK-c-Jun-p53 pathway. **A** Representative images of the apoptosis arrays derived from Gem treated MIA PaCa-2 derived VA or MAGEA2 C10 clones are given (n = 4 dots from 2 independent experiments). Arrows indicate the apoptotic related proteins with significant changes. **B** Western blot analysis of placebo or Gem treated MIA PaCa-2 derived VA and MAGEA2 C10 clones. Hsc70 was used as a loading control. **C** Western blot analysis of placebo -/ + anisomycin (Ani) and Gem-/ + Ani treated MIA PaCa-2 derived VA and MAGEA2 C10 clones. **D**, **E** Bar charts represent the relative survival of MIA PaCa-2/CFPAC-1 derived VA and MAGEA2 clones after treated with Gem or Gem plus Ani (Anisomycin). Means ± S.E.M. (n = 3 independent experiments). *p < 0.05; **p < 0.01; ***p < 0.001; ****p < 0.0001. **A** Student’s *t* test. **D**, **E** One-way ANOVA

We and others have previously shown that MAGEA family members, such as MAGEA2/MAGEA3, can collaboratively regulate signalling pathways [24, 38]. Therefore, we chose MAGEA2 as a representative member to examine the functional role of MAGEA in regulating chemotherapy-induced cell apoptosis. To elucidate this, we performed apoptotic arrays with Gem treated MIA PaCa-2 derived MAGEA2 or VA clones (Fig. 3A). Our array results indicated that in the presence of Gem, MAGEA2 overexpression down-regulated the levels of CD40, CD40L, cytochrome C and FasL in MIA PaCa-2 cells as compared with the empty vector transfected cells (Fig. 3A), while these factors were known to be involved in p53 signalling pathways. Indeed, previous studies showed that JNK (c-Jun N-terminal Kinase) could regulate p53 dependent apoptosis via c-Jun [39]. Additionally, flow cytometry (FACS) analysis indicated that MAGEA2 overexpression prohibited the up-regulation of FasL expression in gemcitabine treated MIA PaCa-2 cells, while there was no difference in its expression between VA and MAGEA2 expressing cells in the absence of gemcitabine (Additional file 4: Fig. S2I). Annexin V-PI apoptosis assays indicated that overexpression of MAGEA2 prohibited gemcitabine induced apoptosis in MIA PaCa-2 cells as compared with the empty vector transfected cells, while there was no difference in apoptosis between MAGEA2 overexpressing cells and VA cells in the absence of gemcitabine (Additional file 4: Fig. S2J). These results suggested that MAGEA2 regulated gemcitabine induced apoptosis. To determine whether MAGEA2 regulated chemotherapy-induced apoptosis via JNK-c-Jun-p53 dependent pathway, we performed western blot analysis of placebo- or Gem-treated MIA PaCa-2 derived MAGEA2/VA clones, indicating that MAGEA2 overexpression prohibited the up-regulation of p-JNK, p-c-Jun and p-p53 expression in MIA PaCa-2 cells after Gem treatment as compared to empty vector transfected cells (Fig. 3B). Consistent with this finding, these proteins were shown to be regulators of Gem-induced apoptosis [39, 40]. Importantly, pharmacological activation of JNK using anisomycin [41] in the MIA PaCa-2 derived MAGEA2 clone rescued Gem-induced up-regulation of p-JNK, p-c-Jun and p-p53 expression in comparison with placebo-treated cells, whereas it had no significant effect on VA control cells (Fig. 3C). Moreover, treatment with anisomycin alone led to a greater up-regulation of p-JNK, p-c-Jun, and p-p53 expression in MAGEA2-expressing MIA PaCa-2 cells compared to VA cells (Fig. 3C). Additionally, anisomycin treatment repressed the proliferation of MAGEA2 expressing MIA PaCa-2/CFPAC-1 cells and rescued their sensitivity to Gem (Fig. 3D, E). However, anisomycin had no significant synergic inhibitory effect with Gem on the survival of VA control cells (Fig. 3D, E). Together, our data show that MAGEA2 can modulate tumor cell resistance to Gem via regulation of the JNK-c-Jun-p53 mediated cell apoptosis.

### Gemcitabine stimulated human pancreatic stellate cells increases GDF15 secretion to further enhance MAGEA2-mediated gemcitabine resistance in tumor cells via a paracrine signalling

**Fig. 4:**
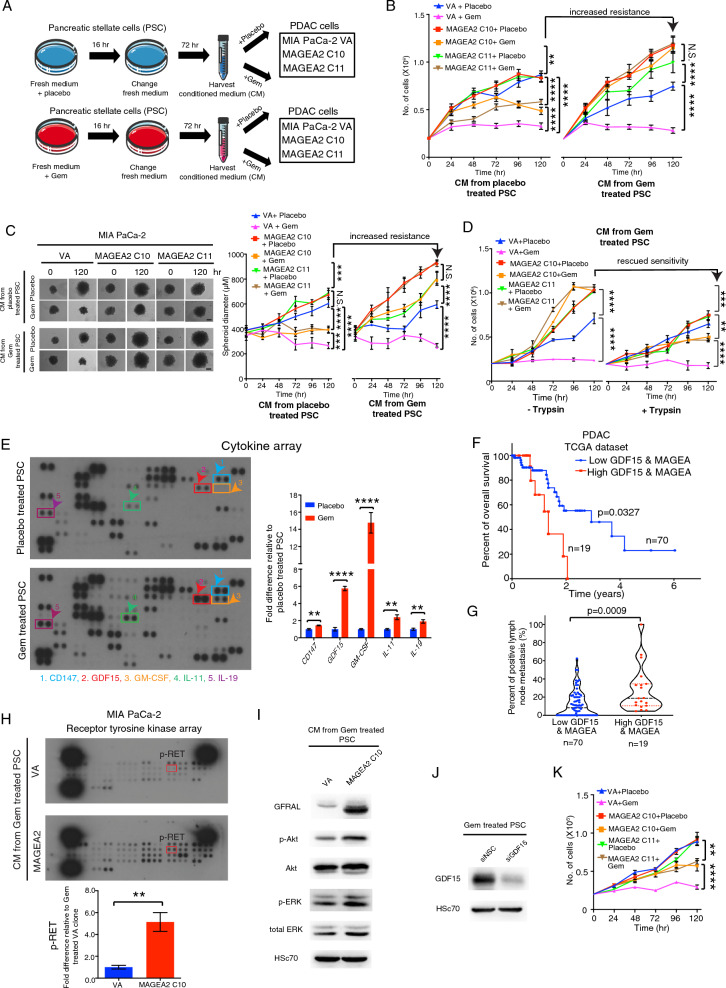
Gemcitabine stimulated human pancreatic stellate cells further enhances the gemcitabine resistance of MAGEA2-expressing tumor cells via a paracrine signalling. **A** Schematic diagram of the experimental design to test the effect of conditioned medium (CM) from placebo/Gem treated PSC in MIA PaCa-2 derived VA and MAGEA2 clones. **B** MIA PaCa-2 derived VA and MAGEA2 C10 clones were exposed with the CM of placebo or Gem treated PSC -/ + Gem for up to 5 days. **C** MAGEA2- or VA-expressing MIA PaCa-2 spheroids were cultured with either the CM of placebo or Gem treated PSC together with placebo or Gem for up to 5 days. **D** The CM harvested from placebo/Gem treated PSCs were first incubated with or without trypsin for 24 h at 37 °C, which were then added to MIA PaCa-2 derived MAGEA2 and VA clones for up to 120 h. **E** Quantitation of the fold difference in cytokine expression between placebo and Gem treated PSCs (n = 4 dots from 2 independent experiments). Representative pictures of the cytokine arrays derived from placebo or Gem treated PSCs. Arrows indicate the cytokines with significant change. **F**, **G** Analysis of TCGA database revealed that high expression of GDF15 and MAGEA was associated with poor overall survival and enhanced lymph node metastases in PDAC patients (n = 89 patients, TCGA dataset). **H** Receptor tyrosine array. Quantification of fold difference in p-RET level between MIA PaCa-2 derived VA and MAGEA2 clones after exposure with the CM of Gem stimulated PSCs. **I** Western blot analysis of MIA PaCa-2 derived VA and A2 C10 clones after treated with the CM of Gem stimulated PSCs. **J** Western blot analysis of PSCs transfected with non-silencing control (siNSC) or GDF15 targeting siRNA molecules for 48 h. **K** Line graph shows the cell growth of MIA PaCa2-MAGEA2 and VA clones after exposed with CM from siGDF15 depleted PSCs in the presence/absence of Gem over time (n = 3 independent experiments). *p < 0.05; **p < 0.01; ***p < 0.001; ****p < 0.0001. **B**–**D**, **K** Two-way ANOVA. **E**, **G**, **H** Student’s *t* test. **F** Log-rank (Mantel-Cox) test. Scale bars in (**C**) represent 200 μm

Recent studies showed that pancreatic stellate cells (PSCs) contributed to chemoresistance and metastasis [10, 31]. To explore their role in MAGEA-mediated gemcitabine resistance, we harvested conditioned medium (CM) from human PSCs after treatment with either placebo or Gem. The CM was then added to MIA PaCa-2 derived MAGEA2 and VA clones in the presence of placebo or Gem (Fig. 4A). Interestingly, exposure to CM from Gem treated PSCs enhanced the proliferation of MAGEA2-expressing MIA PaCa-2 cells compared to the cells exposed with CM from placebo-treated PSCs. However, there was no significant difference in cell proliferation between VA control cells being treated with CM from either Gem or placebo treated PSCs (Fig. 4B). Importantly, MAGEA2-expressing MIA PaCa-2 cells, but not VA control cells, were more resistant to Gem when cultured in the CM from Gem stimulated PSC (Fig. 4B). This observation was further confirmed by our spheroid assay results in (Fig. 4C). To determine whether the protein or metabolite secreted by the Gem stimulated PSC affected the Gem resistance of MAGEA2-expressing tumor cells, we first treated the CM of Gem stimulated PSC with trypsin overnight at 37 °C, and then applied it to the MAGEA2 and VA expressing clones. Results indicated that the trypsin digestion of CM from Gem treated PSC restored the Gem sensitivity of MAGEA2-expressing cells (Fig. 4D), showing that Gem treated PSC controlled the growth of MAGEA2-expressing cell was indeed paracrine signalling proteins in nature. We then conducted a proteome profiler cytokine XL array analysis on placebo-treated and Gemcitabine (Gem)-treated pancreatic stellate cells (PSC). Our results revealed that the expression of several cytokines, including CD147, GDF15 (growth/differentiation factor-15), GM-CSF, IL-11, and IL-19, was significantly up-regulated in Gem-treated PSC compared to placebo-treated PSC (Fig. 4E). Further RT-PCR analysis showed that GDF15 exhibited the most significant increase in expression in Gem-treated PSC, which was also confirmed by western blot analysis (Additional file 5: Fig. S3A, B), indicating its up-regulation in response to Gem treatment.

To exploit the clinical significance of our observation, we performed an analysis of the TCGA dataset showing that high GDF15 expression associated with increased tumor sizes and poor overall survival in PDAC patients (Additional file 5: Fig. S3C, D), while the PDAC patients with high GDF15 and MAGEA expression distinguished into those with worse overall survival and elevated lymph node metastasis (Fig. 4F, G). Importantly, high expression of MAGEA and GDF15 stratified 5′FU treated GC, PDAC or NSCLC patients into those with poor overall/relapse free survival (Additional file 5: Fig. S3E–G). In contrast, the expression of GM-CSF was not correlated with overall survival in NSCLC and PDAC patients (Additional file 5: Fig. S3H, I). Given that GDF15 may affect MAGEA2-mediated chemoresistance by transducing signals into the cells, we performed an unbiased approach using a receptor tyrosine kinase array to analyze MIA PaCa-2 derived MAGEA2 and empty vector transfected clones after exposure to conditioned medium (CM) from Gem treated PSC. Array result indicated that the expression level of p-RET was increased in MAGEA2-expressing MIA PaCa-2 cells (Fig. 4H). Recent studies showed that GDF15 bound and activated its receptor GDNF receptor α-like (GFRAL) to interact with the co-receptor RET, while this interaction subsequently activated Akt and ERK1/2 mediated cell proliferation and inhibited JNK-c-Jun-p53 mediated apoptosis [14, 42]. Further analysis showed that in the presence of CM from Gem treated PSCs, the expression of GFRAL and its downstream effectors p-Akt and p-ERK1/2 was increased in MAGEA2-expressing cells as compared with empty vector transfected cells (Fig. 4I). Remarkably, silencing GDF15 expression in PSCs rescued its paracrine effect on the Gem resistance of MAGEA2-expressing cells (Fig. 4J, K). Together, these data indicated that Gem stimulated PSCs could regulate the chemosensitivity of MAGEA2-expressing tumor cells via the GDF15-GFRAL mediated paracrine signaling.

### MAGEA2-expressing cells promotes gemcitabine resistance and metastasis in an orthotopic PDAC animal model

**Fig. 5:**
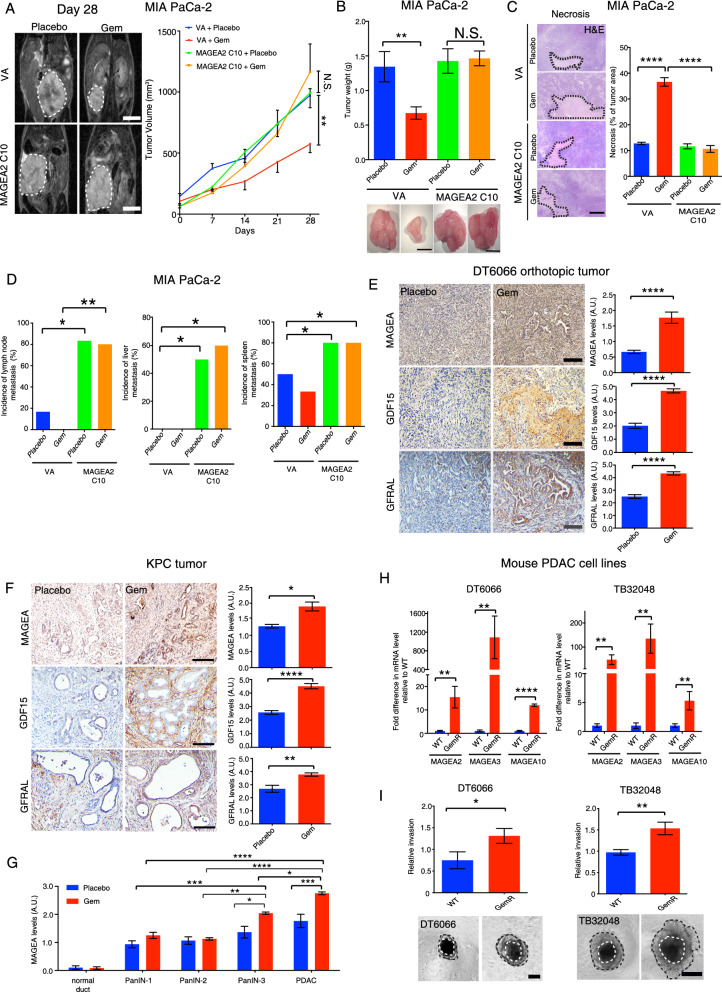
MAGEA2 overexpression confers gemcitabine resistance in vivo. **A** 1 × 10^6^ VA- or MAGEA2-expressing MIA PaCa-2 cells were implanted into the pancreases of female nude mice, while the tumor bearing mice were injected with placebo or 50 mg/kg Gem intraperitoneally once a week for 4 times. Representative MRI images of tumors in each group 28 days post-treatment (n = 6–10 mice per treatment group). **B** Bar charts represent the tumor weight of each treatment group (n = 4–6 tumors per group). Representative gross images of placebo or Gem treated MAGEA2/VA-expressing tumors are given. **C** Representative H&E images of tumors derived from each group are given. Black dot lines indicate the necrotic areas of tumors in each group. Bar chart shows the percentage of necrotic area in each group (n = 4 tumors per group). **D** The incidence of pancreatic metastasis in treated tumor bearing mice (n = 4–6 mice per treatment group). **E**, **F** Immunohistochemical analysis of pan-MAGEA, GDF15 and GFRAL expression in pancreatic tumors derived from DT6066 orthotopic tumors (mouse derived pancreatic cancer cell line) and KPC mice being treated with placebo or gemcitabine (n = 5–6 tumors analyzed per group). **G** Correlation between MAGEA expression level and pancreatic cancer progression in placebo- or Gem-treated KPC mice was measured by counting the percentage of either pancreatic ducts at different stages in intraepithelial neoplasia or invasive areas in PDAC (n = 4–5 tumors analyzed per group). **H** RT-PCR analysis of MAGEA2, MAGEA3, MAGEA10 expression levels in GemR DT6066 or TB32048 (KPC tumor derived cells) cells and their parental wild type (WT) cells (n = 3–12 experimental repeats). **I** Invasion assays showed that GemR DT6066 and TB32048 cells were more invasive in comparison with their parental WT cells (n = 4 experimental repeats). Results are given as means ± S.E.M. *p < 0.05; **p < 0.01; ***p < 0.001; ****p < 0.0001. **A** Two-way ANOVA. **B**, **C**, **G, H** One-way ANOVA. **D** Chi-square test. **E**, **F**, **I** Student’s *t* test. Scale bars in (**A**, **B**) represent 1 cm, (**C**) 200 μm, (**E**) 100 μm, (**F**) 50 μm, (**I**) 200 μm

To examine the effect of MAGEA2 overexpression on chemoresistance in vivo, we orthotopically implanted MAGEA2- or VA-expressing MIA PaCa-2 cells into the pancreases of nude mice, which were then treated with placebo or 50 mg/kg Gem 4 times within 28 days. Magnetic resonance imaging (MRI) indicated that MAGEA2-expressing tumors were resistant to Gem treatment compared to the empty vector transfected tumors (Fig. 5A, B). A decrease in tumor necrosis was also observed in Gem treated MAGEA2-expressing tumors compared to the Gem treated VA control tumors (Fig. 5C). In addition, mice bearing MAGEA2-expressing tumors had higher incidence of metastasis including spleen, liver and inguinal lymph nodes even after Gem treatment (Fig. 5D). Similar to our in vitro finding, we observed up-regulation of GFRAL expression in MAGEA2-expressing tumors compared with the VA control tumors (Additional file 6: Fig. S4A). Our previous studies showed that orthotopic DT6066 (mouse derived pancreatic cancer cell line) and spontaneous KPC (*Kras*^LSL-G12D/+^; *p53*^R172H/+^*;Pdx1-Cre*) tumors were resistant to Gem monotherapy after a long period of treatment, and these treated tumors were highly desmoplastic, demonstrating the regulatory role of pancreatic stellate cells in tumor chemoresistance [31]. To further confirm the role of GDF15-GFRAL paracrine signalling in MAGEA2-mediated Gem resistance, we immunostained tissue sections derived from the placebo or Gem treated DT6066 orthotopic and KPC tumors in our previous study, with pan-MAGEA, GFRAL and GDF15 antibodies respectively. Immunohistochemical analysis indicated that MAGEA, GFRAL and GDF15 expression was up-regulated in both the Gem treated DT6066 and KPC tumors when compared with the placebo treated DT6066 and KPC tumors respectively (Fig. 5E, F). Since pancreatic tumor gradually progresses via morphologically distinct stages, known as PanIN-1, PanIN-2, PanIN-3 and PDAC, we showed a high MAGEA expression correlated with Gem treated KPC tumor progression (Fig. 5G). Due to lack of commercially available murine MAGEA2, MAGEA3 or MAGEA10 antibodies, DT6066 and TB32048 (spontaneous KPC tumor derived cells) cells were chronically exposed to Gem until they became resistant. To confirm our in vivo observation, we performed RT-PCR analysis of the MAGEA family members and GFRAL expression in these resistant cell lines, showing that the expression of MAGEA family members (MAGEA2, MAGEA3, MAGEA10) and GFRAL were up-regulated in both of the GemR cell lines when compared to their parental WT cell lines (Fig. 5H, Additional file 6: Fig. S4B, C). Importantly, the spheroids derived from GemR DT6066 and TB32048 cells were more invasive when compared with their parental WT cells (Fig. 5I). Together, these data show that MAGEA-GDF15 mediated tumor-stromal crosstalk can promote Gem resistance and cancer progression in three different mouse models of PDAC probably by up-regulating GFRAL mediated AKT and ERK1/2 dependent cell survival pathway.

### Multi-MAGEA antigen targeted DNA therapeutic vaccination inhibits the growth of gemcitabine resistant PDAC cells in vivo

**Fig. 6:**
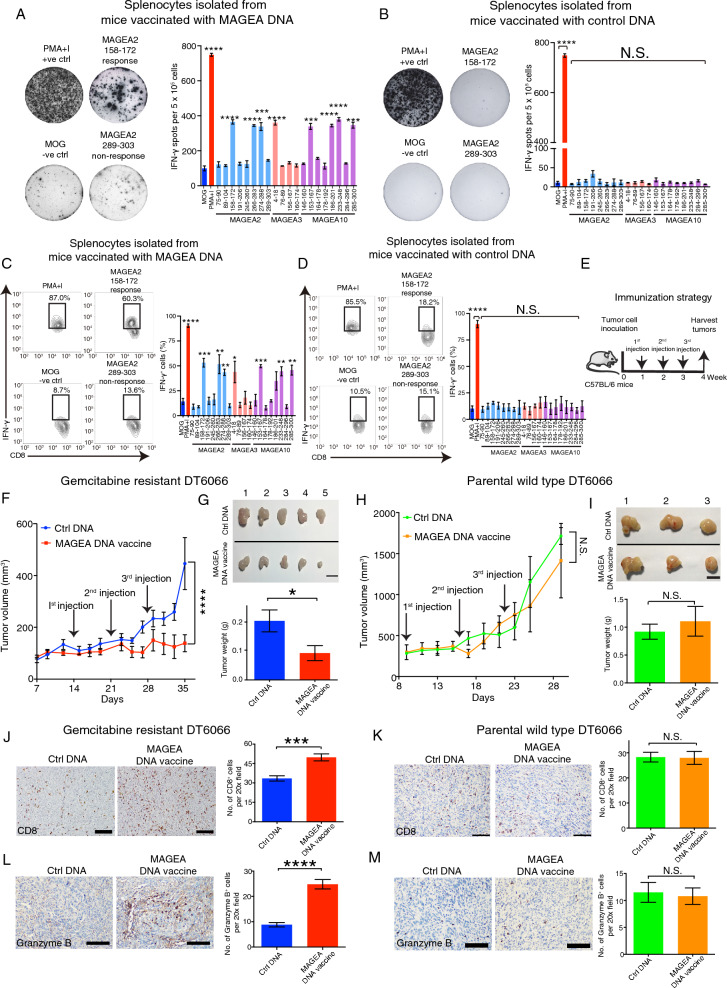
Immunization with multi-MAGEA antigen targeted DNA vaccine significantly represses the growth of gemcitabine resistant DT6066 tumors. **A**–**D** C57BL/6 mice were immunized with either murine control DNA or MAGEA DNA vaccine plus poly (I:C) once per week up to 3 weeks. ELISpot assay (**A**, **B**) and flow cytometry (**C**, **D**) analysis of IFN- γ splenocyte responses to 15–16-mers peptides stimulation after vaccination with either MAGEA DNA vaccine or control DNA. **E** Schematic diagram of the experimental design to examine the effect of multi-MAGEA antigen targeted DNA vaccine in GemR or WT DT6066 tumor bearing mice. **F**, **G** GemR DT6066 tumor size was significantly decreased in mice vaccinated with the MAGEA DNA vaccine compared with the control group (n = 5 tumors per treatment group). **H**, **I** Immunization with MAGEA DNA vaccine had no apparent effect on WT DT6066 tumor growth when compared with the control DNA-vaccinated group. (n = 3 tumors per treatment group). Representative bright field images of the tumors derived from either the MAGEA DNA- or control DNA-vaccinated groups are given. Bar charts represent the tumor weight of the mice vaccinated with MAGEA DNA vaccine or control DNA. **J**–**M** Immunohistochemical staining of CD8 (**J**, **K**) or granzyme B (**L**, **M**) in GemR or WT DT6066 tumors after vaccinated with either MAGEA DNA vaccine or control DNA. Representative images of CD8 or granzyme B immunostaining are given. Bar chart represents mean CD8^+^ /granzyme^+^ T cell number. Bar charts represent means ± S.E.M. *p < 0.05; **p < 0.01; ***p < 0.001; ****p < 0.0001. N.S. no significant difference. **A**–**D** One-way ANOVA. **F**, **H** Two-way ANOVA. **G**, **I**–**M** Student’s *t* test. Scale bars in (**G**, **I**) represents 1 cm, (**J**–**M**) 50 μm

MAGEA3 cancer vaccine has entered several clinical trials for cancer treatment [22, 43]. Unfortunately, this vaccine did not show any patient survival benefit. One of the possible explanations is that these patients were known to express more than one MAGEA family member in their tumors [44]. Due to similarity between MAGEA family members [17, 24], they are likely to compensate each other in functions. Moreover, we have shown that MAGEA2 and MAGEA3 can work alone or together to inhibit p53 activity [24]. Consequently, targeting only one MAGEA family member may be not sufficient to trigger adequate immunization to eliminate MAGEA-expressing cells. Recent studies have demonstrated that the administration of an ovalbumin (OVA) antigen expression plasmid in mice can induce the expression of OVA and effectively stimulate the production of antibodies against OVA [45]. Additionally, other research has shown that the injection of DNA vaccines, specifically plasmids, can trigger robust humoral and cellular immune responses in mice [46]. Therefore, DNA vaccines present a promising approach for developing a single-target vaccine that can simultaneously target all three MAGEA antigens. To investigate the immunogenicity of MAGEA family members, we initially constructed a murine DNA vaccine targeting multiple antigens, namely MAGEA2, MAGEA3, and MAGEA10 (referred to as the MAGEA DNA vaccine). This was accomplished by subcloning the complete cDNA sequences of these antigens into an overexpression vector. Subsequently, we generated 15–16-mer peptide libraries representing the murine MAGEA family members based on the MHC-I subclass expression observed in Gem-resistant (GemR) DT6066 cells and their parental wild-type (WT) cells, as determined by FACS analysis (Additional file 6: Fig. S4D). To assess the immune response elicited by our MAGEA DNA vaccine against MAGEA2-, MAGEA3- or MAGEA10- encoded epitopes, we immunized C57BL/6 mice with the MAGEA DNA vaccine or control DNA in combination with poly(I:C). This immunization was performed once a week for a total of three doses. Splenocytes were then collected from the mice for analysis using the IFN-γ ELISpot assay. The results indicated that immunization with murine MAGEA DNA vaccine in the mice, but not the control DNA, induced an IFN-γ splenocyte response against MAGEA2-, MAGEA3- or MAGEA10-encoded epitopes, whereas there was no immune response observed against the control HER2-encoded epitope, confirming the efficacy and specificity of our immunization strategy (Fig. 6A, B). Furthermore, FACS analysis demonstrated that the immunization of murine MAGEA DNA vaccine in C57BL/6 mice induced a robust IFN-γ CD8^+^ and CD4^+^ T cell response against MAGEA2-, MAGEA3- or MAGEA10-encoded epitopes, but not the control myelin oligodendrocyte glycoprotein (MOG)-encoded epitopes (Fig. 6C, Additional file 7: Fig. S5A), whereas the control DNA did not elicit any MAGEA2, MAGEA3 or MAGEA10 specific CD8^+^ and CD4^+^T cell response (Fig. 6D, Additional file 7: Fig. S5B). To explore the effect of MAGEA DNA vaccination on tumor growth, WT or GemR mouse pancreatic cancer DT6066 cells were injected into C57BL/6 mice subcutaneously, which were then immunized with either MAGEA DNA vaccine or control DNA (Fig. 6E). Results showed that the immunization with MAGEA DNA vaccine repressed the growth of GemR DT6066 tumors as compared with the control group (Fig. 6F, G), whereas it showed no obvious effect on the growth of WT DT6066 tumors (Fig. 6H, I). Furthermore, our IHC analysis indicated that the number of infiltrating CD8^+^ T cells in GemR DT6066 tumors was increased in mice vaccinated with MAGEA DNA compared with mice vaccinated with control DNA (Fig. 6J), whereas the immunization with MAGEA DNA vaccine did not affect CD8^+^ T cell infiltration in WT DT6066 tumors (Fig. 6K). To verify the immunogenicity of our MAGEA DNA vaccine, we detected the activity of infiltrating CD8^+^ T cells by immunostaining with granzyme B antibody. Our IHC data indicated the number of granzyme B positive cells in GemR DT6066 tumors was increased in mice vaccinated with MAGEA DNA vaccine as compared to the mice vaccinated with control DNA, but there was no difference in granzyme B positive cell numbers between WT DT6066 tumors derived from mice vaccinated with either MAGEA DNA vaccine or control DNA (Fig. 6L, M). Notably, treatment with MAGEA vaccination resulted in reduced expression levels of GDF15 and GFRAL in GemR DT6066 tumors compared to the control DNA-treated group, whereas no significant effect on their expression levels was observed in WT DT6066 tumors (Additional file 7: Fig. S5C-F). Importantly, the MAGEA vaccination treatment did not induce noticeable changes in mouse body weight or alter the microscopic architecture of multiple organs (Additional file 7: Fig. S5G, H). These data validate the immunogenicity, specificity, and safety of the MAGEA DNA vaccine against GemR cells that express high levels of MAGEA2, MAGEA3, and MAGEA10.

Due to the fact that cancer patients often developed simultaneous resistance to multiple chemotherapeutic drugs [47], we examined whether this was the case in our study. Interestingly, RT-PCR analysis indicated that human 5′FU resistant PDAC and NSCLC cell lines had an increased MAGEA2, MAGEA3 and MAGEA10 expression in comparison with their parental WT cells (Additional file 8: Fig. S6A-D). Remarkably, IC50 experiments showed that human/mouse Gem or 5′FU resistant PDAC or/and NSCLC cell lines were cross-resistant to Gem and 5′FU treatment (Additional file 8: Fig. S6E-J). These findings support that immunization with multi-MAGEA antigen engineered DNA vaccine may potentially treat multiple drug resistance in cancers.

**Fig. 7:**
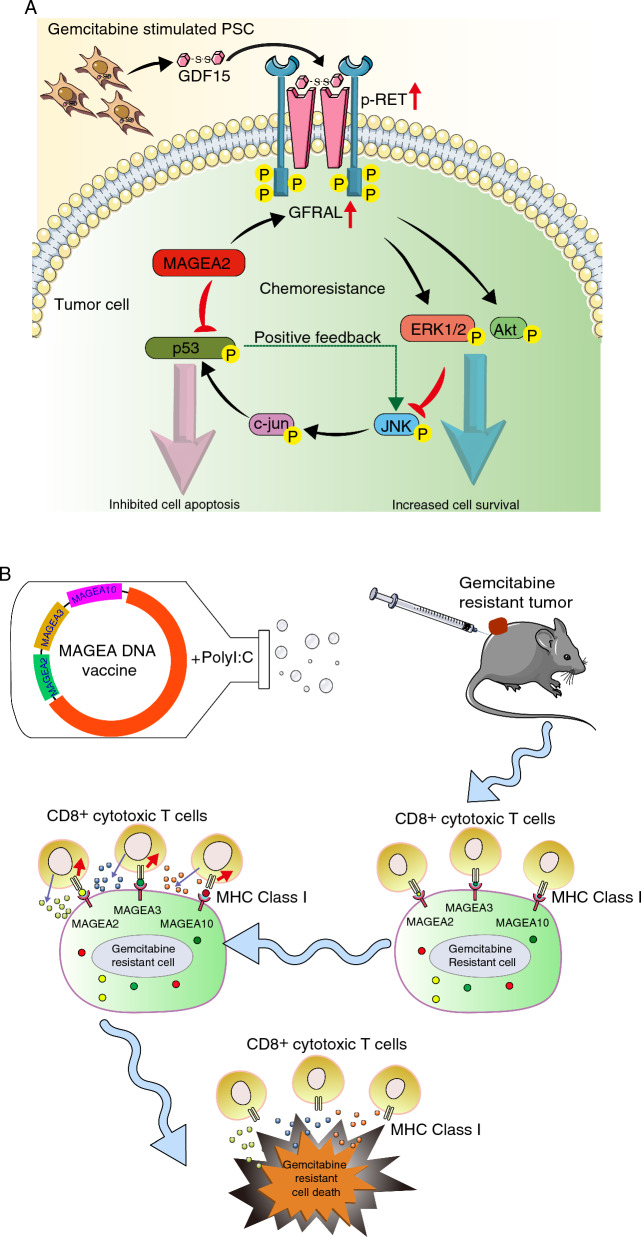
Schematic representation of the molecular mechanism of MAGEA2-mediated paracrine control of chemoresistance as well as targeting MAGEA as an effective immunotherapy against GemR PDAC cells. **A** Expression of MAGEA family members, including MAGEA2, MAGEA3 and MAGEA10, is up-regulated in GemR PDAC cell lines and patient derived organoids. Upon Gem treatment, MAGEA2 inhibits Gem-induced JNK-c-Jun-p53 dependent apoptosis by repressing p53 mediated positive feedback loop with JNK activation. Importantly, Gem treatment stimulates PSCs to secrete GDF15 to activate its receptor GFRAL, expression of which is significantly up-regulated in MAGEA2-expressing tumor cells compared to the VA control cells. The GDF15-GFRAL interaction triggers the recruitment and activation of its co-receptor RET, which then increases the activity of its downstream Akt and ERK1/2 mediated cell survival pathway. Furthermore, the GDF15-GFRAL axis can potentially inhibit JNK-c-Jun-p53 mediated apoptosis via activation of the Akt and ERK1/2 dependent pathway. As a result, MAGEA2-mediated tumor-stromal crosstalk controls cancer chemoresistance. **B** Targeting multiple MAGEA family members with DNA vaccine elicits a robust CD8^+^ T cell response against gemcitabine resistant PDAC cells expressing high levels of MAGEA2, MAGEA3 and MAGEA10

Overall, our findings indicate that MAGEA2 plays a crucial role in tumor-stromal crosstalk, which controls chemoresistance and metastasis by regulating both the GFRAL-RET mediated Akt and ERK1/2 dependent survival pathway, and the JNK-c-Jun-p53 mediated apoptosis pathway. These data establish new molecular mechanisms of chemoresistance in vivo, that PSC-derived paracrine signalling directly affects tumor chemoresistance regulated by MAGEA antigens. Importantly, targeting MAGEA antigens with our DNA vaccine elicits robust immune responses to eliminate chemoresistant cancer cells, indicating the potential role of MAGEA antigens as determinants of effective immunotherapy in pancreatic cancer treatment (Fig. 7).

## Discussion

Our data establish that MAGEA family members are not just immunogenic antigens, but also function as regulators of tumor-stromal crosstalk mediated cancer chemoresistance and metastasis. These results imply us to reconsider the role of MAGEA antigen-mediated paracrine network in the control of pancreatic cancer chemoresistance.

We and other researchers have demonstrated that MAGEA regulates tumor cell survival, autophagy and apoptosis through mechanisms that are not fully understood. Additionally, it has been shown that MAGEA family members can cooperatively regulate cellular pathways together [24, 26, 27]. However, their cellular role in regulating cancer chemoresistance and progression has not been fully explored until this study. Our observations regarding the cooperative role of MAGEA2, MAGEA3, and MAGEA10 in regulating PDAC chemoresistance align with previous studies [24, 38]. To gain further insight into the underlying mechanism, we selected MAGEA2 as a representative member to investigate its regulatory role in determining cancer chemoresistance. Our current study further indicates that MAGEA2 expression in tumor cells inhibits the phosphorylation of JNK and its downstream effectors c-Jun and p53 to regulate tumor chemoresistance and metastasis. Since p53 participates in a positive feedback mechanism with JNK to regulate cancer progression and metastasis [39, 48], MAGEA2 may repress JNK activity via inhibition of the p53-mediated positive feedback loop with JNK. Indeed, our rescue experiment shows that pharmacological activation of JNK by anisomycin prohibits the proliferation of MAGEA2-expressing cell and restores its sensitivity to Gem, suggesting that combined anisomycin and Gem can be a novel treatment method to overcome MAGEA-mediated Gem resistance in cancers.

Accumulating evidence suggests that intracellular signalling between cancer cells and stromal cells within the tumor microenvironment dictates chemoresistance and cancer progression [31]. Indeed, activated PSCs have been shown to secrete chemokines/cytokines which enhance cancer cell survival to promote chemoresistance [11]. Given the important role of PSCs in PDAC chemoresistance, inhibiting PSC activity via the blockade of sonic hedgehog (SHH) pathway was developed, which showed promising effects in multiple preclinical studies [49]. Unfortunately, combinatorial therapy of SHH antagonists and Gem for metastatic PDAC patients failed during a phase II clinical trial [50]. Moreover, PSC elimination has been recognized as another promising approach to improve Gem efficacy in PDAC [51], whereas depleting PSC in the PDAC animal models enhanced Gem resistance and cancer progression [52]. Together, these results indicate that the current inability to identify the key tumor-stromal crosstalk pathway has stopped us from improving the Gem-based treatment of cancer patients. In this study, we discover that after exposure with the CM of Gem stimulated PSC, it can further enhance MAGEA2-expressing tumor cell resistance to Gem. Based on proteomics profiler cytokine array analysis, we show that Gem treatment, instead of inhibiting, stimulates PSC to increase a stress responsive cytokine GDF15 production, which has been proved to regulate chemoresistance and metastasis in multiple cancers [42, 53]. Using both orthotopic and spontaneous models of PDAC, we observe an upregulation of stromal GDF15 and its newly identified receptor tumor-GFRAL expression, as well as MAGEA expression in Gem treated tumors. Importantly, the clinical significance of our discovery is supported by the elevated levels of GDF15 and MAGEA expression in human PDAC patients as well as 5′FU treated GC/NSCLC patients with poor prognosis. Further mechanistic study reveals that stable MAGEA2-expressing cancer cells highly express GFRAL, while exposure of these cells with CM from Gem treated PSC activate the GFRAL-RET mediated Akt and ERK1/2 dependent cell survival pathway to further enhance their Gem resistance. Indeed, we show that silencing GDF15 expression in PSC rescues its effect on MAGEA2-mediated chemoresistance in tumor cells. Consistently, recent studies have shown that the GDF15-GFRAL axis has an important role in regulating energy homeostasis [14] and JNK-c-Jun-p53 mediated apoptotic pathway via the Akt and ERK1/2 mediated signalling pathway [53], suggesting that GDF15 may work together with MAGEA2 to inhibit Gem-induced cell apoptosis too. These unexpected results may explain the failure of SHH inhibitor and Gem combination therapy in the clinics, as the Gem treatment could counteract the inhibitory effect of SHH inhibitor on PSC activity by promoting it to secrete more GDF15.

Immune checkpoint inhibitors have demonstrated limited clinical benefits in PDAC compared to other cancers [54]. While therapeutic DNA cancer vaccination approaches targeting tumor associated antigens have been explored as a promising strategy, especially for patients unresponsive to immune checkpoint inhibitors [55, 56], these approaches have yielded limited patient responses in clinical trials, particularly in the context of PDAC. This is probably due to several reasons including: lack of biomarkers; an immune evasion from a single antigen targeted vaccination; a poorly designed combination strategy with chemotherapy; and weak CD8^+^ T cell response. Unlike previous DNA vaccination approach [20], we designed a DNA vaccine using the full-length cDNA of each MAGEA family member (MAGEA2, MAGEA3 and MAGEA10) instead of just the consensus cDNA sequences between them, thereby avoiding the possibility of missing key immunogenic epitopes from each antigen. Indeed, our MAGEA DNA vaccine successfully elicits effective T cell activities against each antigen and effectively eliminates GemR tumor cells expressing high levels of MAGEA2, MAGEA3, and MAGEA10 in two distinct mouse models of pancreatic cancer. Considering the shared cellular functions among MAGEA family members [24], targeting multiple MAGEA antigens simultaneously offers a potential strategy to prevent immune evasion and tumor relapse during treatment. Furthermore, it is intriguing to propose that PDAC patients who are resistant to gemcitabine and exhibit high MAGEA expression may derive benefits from the MAGEA DNA vaccination approach. However, further investigation is necessary to explore this possibility and establish its efficacy.

Overall, our data strongly indicate that MAGEA-mediated paracrine network confers chemoresistance and determines the efficacy of immunotherapy in cancers. An immunization with multi-MAGEA antigen targeted DNA vaccine can potentially eliminate chemoresistant tumor cells. Our findings create a new field of research where exploiting multi-tumor associated antigen targeted DNA vaccination strategy may be an improved treatment method for PDAC patients.

## Conclusions

In conclusion, our work highlights the pathogenic role of MAGEA2-mediated tumor-stromal crosstalk in PDAC chemoresistance and progression, supporting that targeting multiple MAGEA antigens with a DNA vaccine in one goal can eliminate chemoresistant PDAC tumor cells, and provide the first promising immunotherapy for chemoresistant pancreatic cancer.

### Supplementary Information

**Additional file 1: Table S1**. The list of primer sequences for RT-PCR used in this study.

**Additional file 2: Table S2**. The peptide sequences of MAGEA2/MAGEA3/MAGEA10- or MOG-encoded epitopes.

**Additional file 3: Fig. S1**. Correlation analysis between MAGEA/MAGEB/MAGEC family member expression and patient survival in patients with PDAC. The correlation between MAGEA/B/C member expression and patient survival in PDAC was examined by using the KM plotter database tool. (**A**-**K**) Correlation analysis between MAGEA family member expression and patient survival in PDAC. (**L**–**U**) Association between MAGEB family member expression and patient survival in PDAC. (**V**–**X**) Correlation analysis between MAGEA family member expression and patient survival in PDAC (n = 156 PDAC patients). (**Y**) Association between MAGEA family member expression and patient survival in PDAC (n = 174 patients, TIMER2.0 database). (**A**–**Y**) Log-rank (Mantel-Cox) test.

**Additional file 4: Fig. S2**. MAGEA expression regulates gemcitabine resistance in PDAC cell lines. (**A**) Western bolt analysis of lysates from CFPAC-1 stable clones expressing MAGEA2 (C3 and C13), MAGEA3 (C14 and C17) or MAGEA10 (C7 and C12) plus the vector alone (VA) control line. IC50 experiments showed that MAGEA2/MAGEA3/MAGEA10 expressing clones were more resistant to gemcitabine when compared with the VA control clone (n = 9 experimental repeats). (**B**) Bar charts show the proliferation rate of MIA PaCa-2 or CFPAC-1-stable clones expressing MAGEA2, MAGEA3 or MAGEA10 relative to VA clones in normal culturing medium (n = 6 experimental repeats). (**C**) T3M4 cells were transiently transfected with non-silencing control (siNSC) or targeting MAGEA2, MAGEA3 or MAGEA10 siRNA molecules. After 24 h, transfected cells were split into 96-well plates and treated with a range of gemcitabine (0-2000 nM) (n = 6 experimental repeats). (**D**) Spheroid assay of T3M4 cells transfected siNSC or siMAGEA2/A3/A10 molecules. Bar charts represent means ± S.E.M. (n = 9 experimental repeats). (**E**–**H**) RT-PCR analysis (E, G) and CCK8 assays (F, H) were performed on Capan-1 or T3M4 cells transfected with either non-silencing control siRNA (siNSC) or MAGEA2/MAGEA3/MAGEA10 targeting siRNAs, either transfected alone or in combination as indicated in the figure, in the presence or absence of Gem. Bar charts display the relative mRNA expression levels of MAGEA2, MAGEA3, and MAGEA10, or cell survival across different groups, after normalization to the siNSC-transfected control (ctrl) or the placebo-treated group (n = 3 experimental repeats). (**I**) FACS analysis was performed to evaluate the expression of FasL in MAGEA-expressing MIA PaCa-2 and VA cells, both treated with and without Gem. (**J**) Annexin V-PI apoptosis assays were conducted on MAGEA2 expressing cells and empty vector transfected cells treated with either placebo or gemcitabine. Representative FACS images were obtained, with the red number indicating the percentage of apoptotic cells in each respective group. *p < 0.05; **p < 0.01; ***p < 0.001; **** p < 0.0001. N.S. non-significant difference. (**B**, **D**, **E**–**H**, **J**) One-way ANOVA.

**Additional file 5: Fig. S3**. High GDF15 and MAGEA expression correlates strongly with poor prognosis in different cancers. (**A**) RT-PCR analysis of indicated cytokines in placebo or Gem treated PSC. (**B**) Western blot analysis of GDF15 expression in placebo or Gem treated PSC. (**C**, **D**) Analysis of TCGA database to study the relationship between GDF15 expression and tumor size/overall survival in human PDAC patients (n = 127–140 patient samples). Each sample on the violin plots represents individual patient data. (**E**–**G**) High expression of GDF15 and MAGEA associated strongly with poor overall survival/relapse free survival in 5′FU treated GC, PDAC and NSCLC patients (n = 152 GC patients; n = 69 PDAC patients; n = 1925 NSCLC patients, all from KM plotter database). (**H**, **I**) Association between GM-CSF expression and patient survival in NSCLC or PDAC (n = 980 NSCLC patients. n = 127 PDAC patients, all from TCGA database). *p < 0.05; **p < 0.01. (**A**, **C**) Student’s *t* test. (**D**–**I)** Log-rank (Mantel-Cox) test.

**Additional file 6: Fig. S4**. MAGEA2 expression up-regulates the expression of GFRAL in human and murine PDAC cells both in vitro and in vivo*.* (**A**) VA- or MAGEA2-expressing tumor sections were immunohistochemically stained with MAGEA2 or GFRAL antibody. Representative immunohistochemical stained sections of MAGEA2 or GFRAL are given. Bar chart represents mean staining scores ± S.E.M. (n = 5–6 tumors per group). (**B**, **C**) RT-PCR analysis of GFRAL expression level in gemcitabine resistant (GemR) DT6066 or TB32048 cells and their parental wild type (WT) cells. Means ± S.E.M are given. (n = 3 experimental repeats). (**D**) FACS analysis of the expression of MHC-1 subclass on gemcitabine resistant and their parent wild type DT6066 cells respectively. *p < 0.05; ***p < 0.001. (**A**–**C**) Student’s *t* test. Scale bar in (**A**) represents 100 μm.

**Additional file 7: Fig. S5**. Vaccination with multi-MAGEA antigen targeted DNA vaccine in WT C57BL/6 mice does not cause any adverse side effect. (**A**, **B**) Flow cytometry analysis of IFN- γ CD4^+^ T cell response to 15–16-mers peptides stimulation after vaccination with either MAGEA DNA vaccine or control DNA (n = 3 mice per group). (**C**–**F**) Representative IHC analysis of GDF15 (C, D) and GFRAL (E, F) staining on tissue sections from either GemR or WT tumors in each treatment group (n = 3 tumors analyzed per group). (**G**) Mouse body weights of control empty vector or MAGEA DNA vaccine treated mice. (**H**) Representative H&E stained sections of lung, heart, liver and spleen from the mice being immunized with either MAGEA DNA vaccine or control DNA plus poly I:C. No gross morphological defects were observed. Bar charts represent means ± S.E.M. *p < 0.05; **p < 0.01; ***p < 0.001. ****p < 0.0001. N.S. no significant difference. (**A**, **B**) One-way ANOVA. (**G**) Student’s *t* test. Scale bars in (**C**–**F**) represents 50 µm, (**H**) 200 µm.

**Additional file 8: Fig. S6**. Gemcitabine or 5′FU resistant PDAC and NSCLC cell lines are cross-resistant to 5′FU and gemcitabine treatment. (**A**–**D**) RT-PCR analysis of the MAGEA expression in human 5′FU resistant PDAC cell (**A**, **B**) and NSCLC cells (**C**, **D**) respectively. (**E**–**H**) Human gemcitabine- or 5′FU-resistant PDAC cells (**E**, **F**) and NSCLC cells (**G**, **H**) were cross resistant to 5′FU and gemcitabine as compared with their parental wild type cells. (**I**–**J**) 5′FU or gemcitabine IC50 analysis of mouse gemcitabine- or 5′FU-resistant DT6066 cells and their parental wild type cells (n = 3 independent experiments). Bar charts represent means ± S.E.M. *p < 0.05; **p < 0.01; ***p < 0.001. (**A**–**D**) One-way ANOVA.

## Data Availability

The data used to support the findings of our study are available upon reasonable request.

## Abbreviations

MAGEA: Melanoma associated antigen
PDAC: Pancreatic ductal adenocarcinoma
PSC: Pancreatic stellate cells
GDF15: Growth/differentiation factor 15
GFRAL: GDNF family receptor a-like
RET: Rearranged during transfection
Gem: Gemcitabine
5′FU: Fluorouracil
