# Supplementary material for: Immunization with a multi-antigen targeted DNA vaccine eliminates chemoresistant pancreatic cancer by disrupting tumor-stromal cell crosstalk
Source: J Transl Med. 2023 Oct 9;21:702. doi: 10.1186/s12967-023-04519-3 (PMC10561406; doi:10.1186/s12967-023-04519-3)
Supplement: Supplementary file 1 — Additional file 1: Table S1. The list of primer sequences for RT-PCR used in this study. [file 12967_2023_4519_MOESM1_ESM.docx]

**Additional file 1: table 1. The list of primer sequences for RT-PCR used in this study.**

| Gene name | Direction | Primer sequence |
| --- | --- | --- |
| M-MAGEA2 | Forward: | ACACCCAATACTGCAGCCTC |
|  | Reverse: | GAGGACTCTGGGGAGGACTT |
| M-MAGEA3 | Forward: | GGCAGGTGCCCAATAGTGAT |
|  | Reverse: | AGGGTAGGCTCTGGGATGAG |
| M-MAGEA10 | Forward: | GCTCCTCCTGCACTGCTATG |
|  | Reverse: | GCTGACTGGCTCCTGCATAA |
| M-GFRAL | Forward: | CCACTTGCCTCAGTGTAATTCA |
|  | Reverse: | TGCCTAACATGCTAATGCAGG |
| M-β-actin | Forward: | AAATCTGGCACCACACCTTC |
|  | Reverse: | GGGGTGTTGAAGGTCTCAAA |
| H-MAGEA2 | Forward: | AGCCTCCAGCTTCTCGACTA |
|  | Reverse: | CTGATGGGGACCACTTCCAC |
| H-MAGEA3 | Forward: | TCACCAGATCCTCCCCAGAG |
|  | Reverse: | CGACACTCCCCAGCATTTCT |
| H-MAGEA10 | Forward: | GGGGCTGTATGATGGGATGG |
|  | Reverse: | TGGTGGCAATTCTGTCCTGG |
| H-GDF15 | Forward: | ACCTGCACCTGCGTATCTCT |
|  | Reverse: | CGGACGAAGATTCTGCCAG |
| H-GM-CSF | Forward: | ACTTCCTGTGCAACCCAGATT |
|  | Reverse: | CTCATCTGGCCGGTCTCAC |
| H-CD147 | Forward: | GGAACTCTTCCTGAGGTGGC |
|  | Reverse: | TGATGGGAATCTACGGGGTG |
| H-IL-11 | Forward: | GGGGACATGAACTGTGTTTGC |
|  | Reverse: | GAGGGTCTGGGGAAACTCG |
| H-IL-19 | Forward: | AGAGGAGACACAAGGAGCAG |
|  | Reverse: | TGTCCTTAGCTTGGATGGCTC |
| H-β-actin | Forward: | AAATCTGGCACCACACCTTC |
|  | Reverse: | GGGGTGTTGAAGGTCTCAAA |

H stands for human

M stands for Mouse
