# Supplementary material for: Immunization with a multi-antigen targeted DNA vaccine eliminates chemoresistant pancreatic cancer by disrupting tumor-stromal cell crosstalk
Source: J Transl Med. 2023 Oct 9;21:702. doi: 10.1186/s12967-023-04519-3 (PMC10561406; doi:10.1186/s12967-023-04519-3)
Supplement: Supplementary file 2 — Additional file 2: Table S2. The peptide sequences of MAGEA2/MAGEA3/MAGEA10- or MOG-encoded epitopes. [file 12967_2023_4519_MOESM2_ESM.docx]

**Additional file 2: table 2. The peptide sequences of MAGEA2/MAGEA3/MAGEA10- or MOG-encoded epitopes.**

| Gene name | peptide positions | Peptide sequence |
| --- | --- | --- |
| MAGEA2 | p245-260 | YRQVPNSDPPSYGFLW |
|  | p289-303 | PEKYAEALQDEIDRT |
|  | p158-172 | PSVHSYILVTALGIT |
|  | p191-206 | AVLSVIFMKGNYVSEE |
|  | p89-104 | LYLLHNAQNTKVYDLV |
|  | p266-283 | AETSKMKVLQFFASINKT |
|  | p274-288: | LQFFASINKTHPRAY |
|  | p75-90 | EEASIKGSGGLEDPLY |
| MAGEA3 | p4-18 | SHNTQYCNLEESAQA |
|  | p76-89 | EASIKGSEGLEDPLH |
|  | p156-167 | VDSSVHTYMLVTALGI |
|  | p160-174 | VHTYMLVTALGITYD |
| MAGEA10 | p233-248 | DMVWSILNNIGLYAG |
|  | p153-167 | QDHFAVIFREALECM |
|  | p285-300 | RAYAETTKMKILTFLT |
|  | p146-160 | SNIIRNYQDHFAVIF |
|  | p186-201 | ILTIALELTYDGMMTD |
|  | p164-178 | LECMQLVFGLELKEI |
|  | p178-192: | IDPASHTYILTIAL |
|  | p296-284 | HVPGSNPPFYEFLWGP |
| MOG | p35–55 | MEVGWYRSPFSRVVHLYRNGK. |
