# Supplementary figures and images for: Immunization with a multi-antigen targeted DNA vaccine eliminates chemoresistant pancreatic cancer by disrupting tumor-stromal cell crosstalk
Source: J Transl Med. 2023 Oct 9;21:702. doi: 10.1186/s12967-023-04519-3 (PMC10561406; doi:10.1186/s12967-023-04519-3)

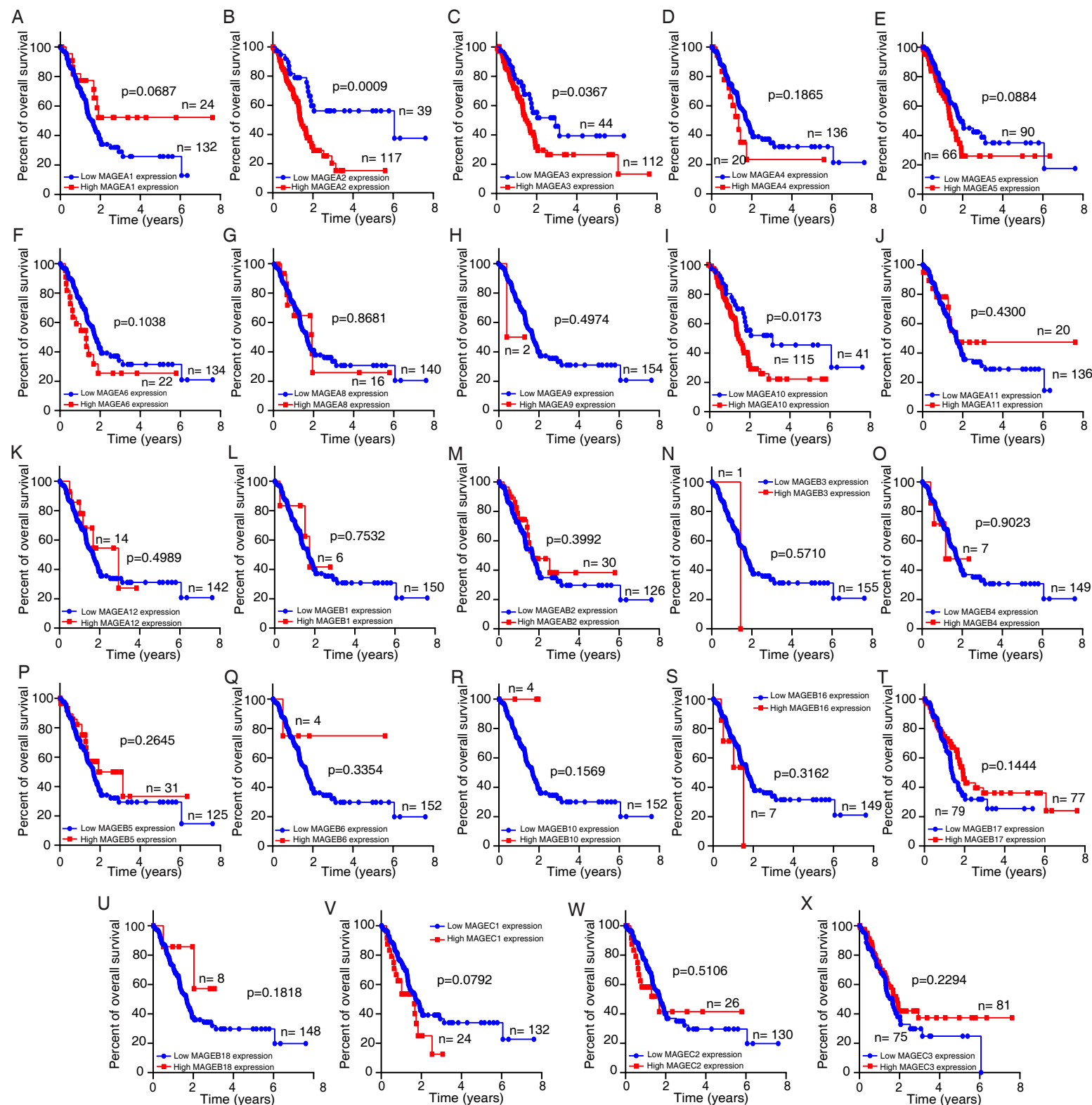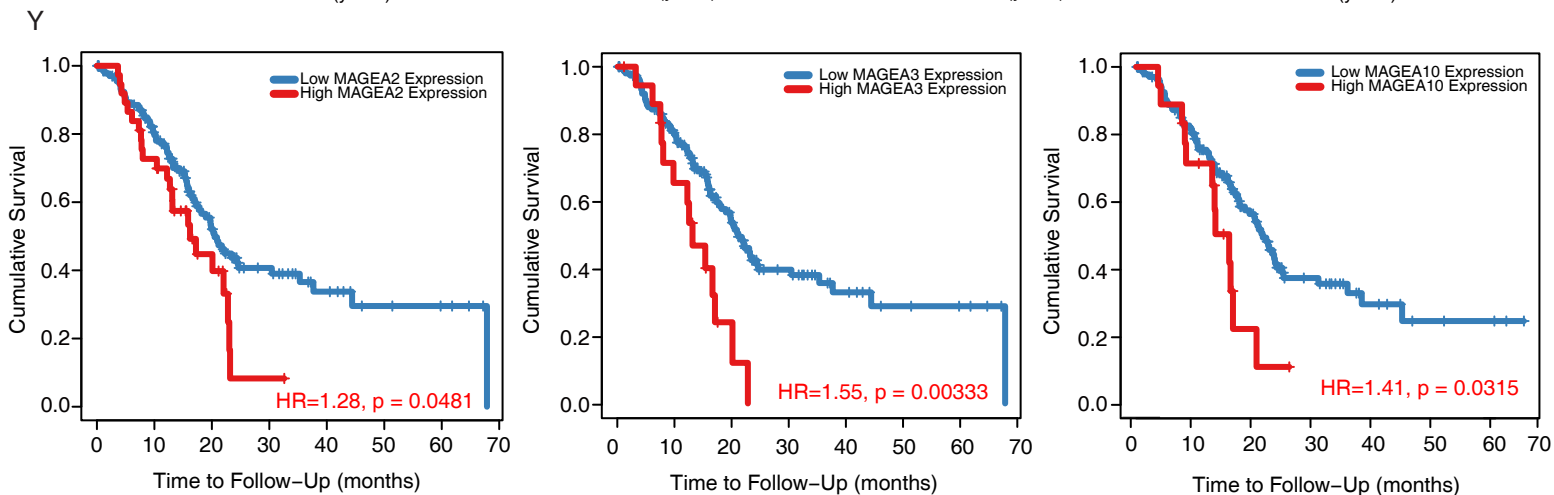

Supplement: Supplementary file 3 — Additional file 3: Fig. S1. Correlation analysis between MAGEA/MAGEB/MAGEC family member expression and patient survival in patients with PDAC. The correlation between MAGEA/B/C member expression and patient survival in PDAC was examined by using the KM plotter database tool. (A-K) Correlation analysis between MAGEA family member expression and patient survival in PDAC. (L–U) Association between MAGEB family member expression and patient survival in PDAC. (V–X) Correlation analysis between MAGEA family member expression and patient survival in PDAC (n = 156 PDAC patients). (Y) Association between MAGEA family member expression and patient survival in PDAC (n = 174 patients, TIMER2.0 database). (A–Y) Log-rank (Mantel-Cox) test. [file 12967_2023_4519_MOESM3_ESM.pdf]

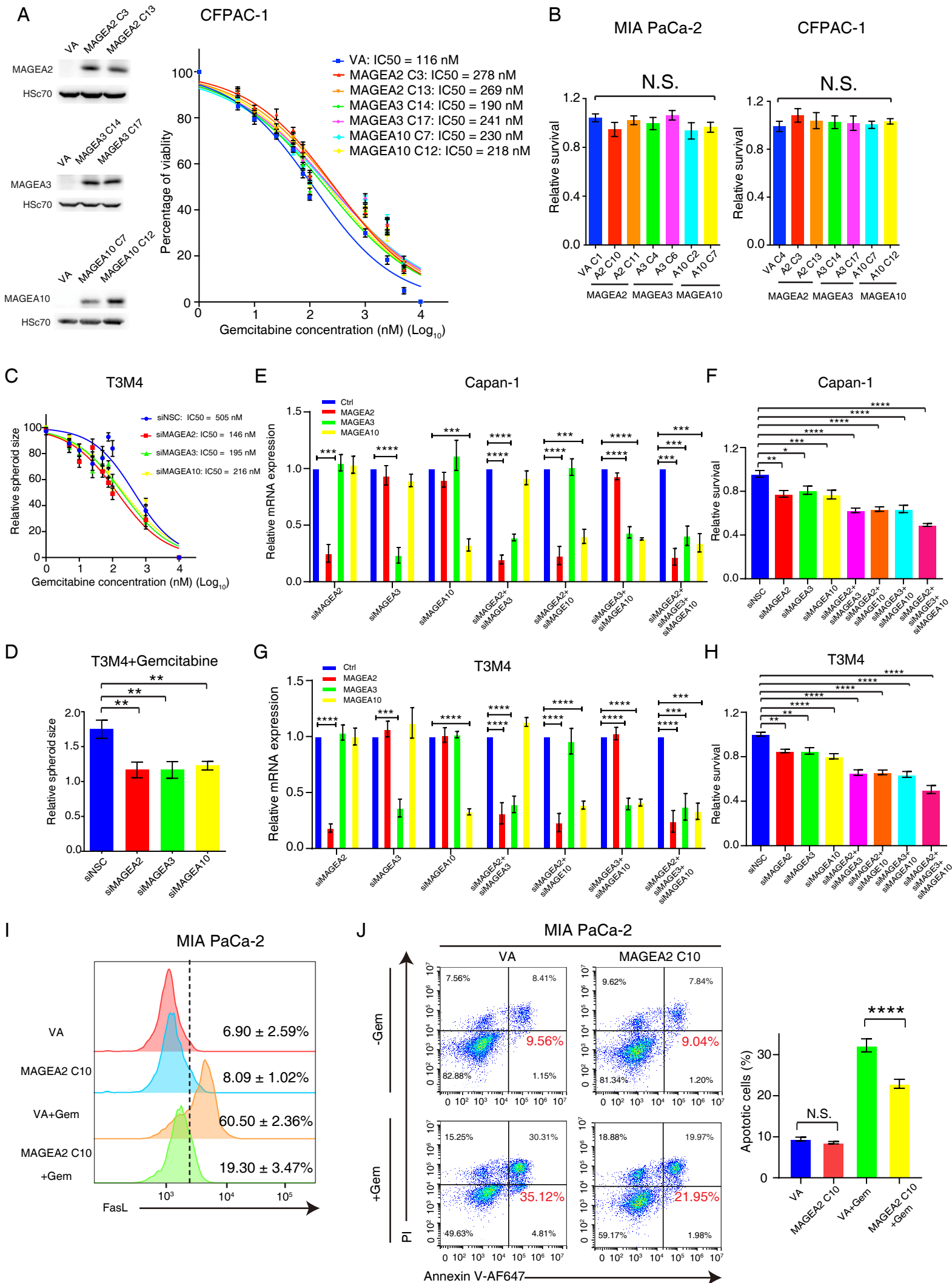

Supplement: Supplementary file 4 — Additional file 4: Fig. S2. MAGEA expression regulates gemcitabine resistance in PDAC cell lines. (A) Western bolt analysis of lysates from CFPAC-1 stable clones expressing MAGEA2 (C3 and C13), MAGEA3 (C14 and C17) or MAGEA10 (C7 and C12) plus the vector alone (VA) control line. IC50 experiments showed that MAGEA2/MAGEA3/MAGEA10 expressing clones were more resistant to gemcitabine when compared with the VA control clone (n = 9 experimental repeats). (B) Bar charts show the proliferation rate of MIA PaCa-2 or CFPAC-1-stable clones expressing MAGEA2, MAGEA3 or MAGEA10 relative to VA clones in normal culturing medium (n = 6 experimental repeats). (C) T3M4 cells were transiently transfected with non-silencing control (siNSC) or targeting MAGEA2, MAGEA3 or MAGEA10 siRNA molecules. After 24 h, transfected cells were split into 96-well plates and treated with a range of gemcitabine (0-2000 nM) (n = 6 experimental repeats). (D) Spheroid assay of T3M4 cells transfected siNSC or siMAGEA2/A3/A10 molecules. Bar charts represent means ± S.E.M. (n = 9 experimental repeats). (E–H) RT-PCR analysis (E, G) and CCK8 assays (F, H) were performed on Capan-1 or T3M4 cells transfected with either non-silencing control siRNA (siNSC) or MAGEA2/MAGEA3/MAGEA10 targeting siRNAs, either transfected alone or in combination as indicated in the figure, in the presence or absence of Gem. Bar charts display the relative mRNA expression levels of MAGEA2, MAGEA3, and MAGEA10, or cell survival across different groups, after normalization to the siNSC-transfected control (ctrl) or the placebo-treated group (n = 3 experimental repeats). (I) FACS analysis was performed to evaluate the expression of FasL in MAGEA-expressing MIA PaCa-2 and VA cells, both treated with and without Gem. (J) Annexin V-PI apoptosis assays were conducted on MAGEA2 expressing cells and empty vector transfected cells treated with either placebo or gemcitabine. Representative FACS images were obtained, with the red number indicating [file 12967_2023_4519_MOESM4_ESM.pdf]

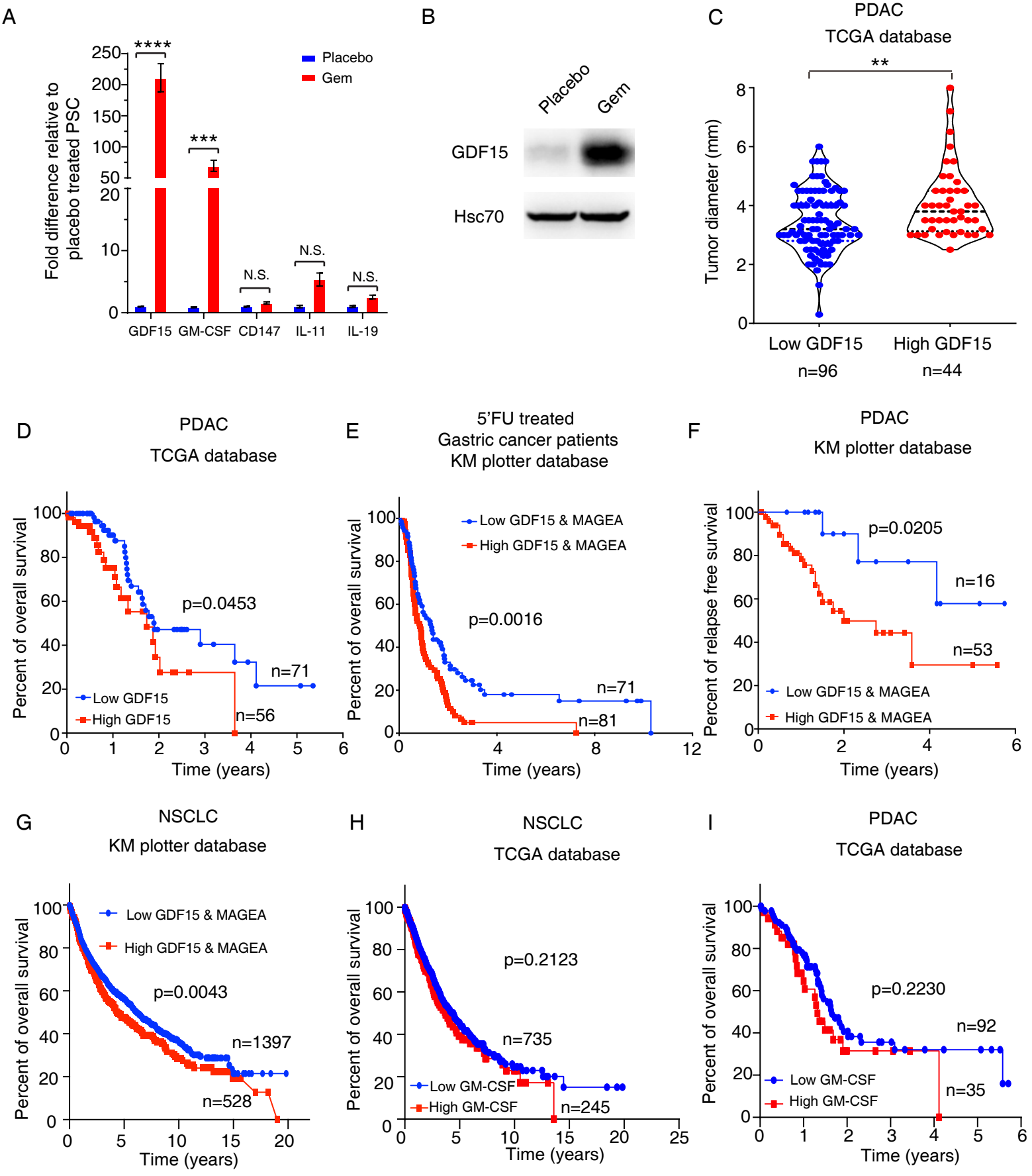

Supplement: Supplementary file 5 — Additional file 5: Fig. S3. High GDF15 and MAGEA expression correlates strongly with poor prognosis in different cancers. (A) RT-PCR analysis of indicated cytokines in placebo or Gem treated PSC. (B) Western blot analysis of GDF15 expression in placebo or Gem treated PSC. (C, D) Analysis of TCGA database to study the relationship between GDF15 expression and tumor size/overall survival in human PDAC patients (n = 127–140 patient samples). Each sample on the violin plots represents individual patient data. (E–G) High expression of GDF15 and MAGEA associated strongly with poor overall survival/relapse free survival in 5′FU treated GC, PDAC and NSCLC patients (n = 152 GC patients; n = 69 PDAC patients; n = 1925 NSCLC patients, all from KM plotter database). (H, I) Association between GM-CSF expression and patient survival in NSCLC or PDAC (n = 980 NSCLC patients. n = 127 PDAC patients, all from TCGA database). *p < 0.05; **p < 0.01. (A, C) Student’s t test. (D–I) Log-rank (Mantel-Cox) test. [file 12967_2023_4519_MOESM5_ESM.pdf]

A

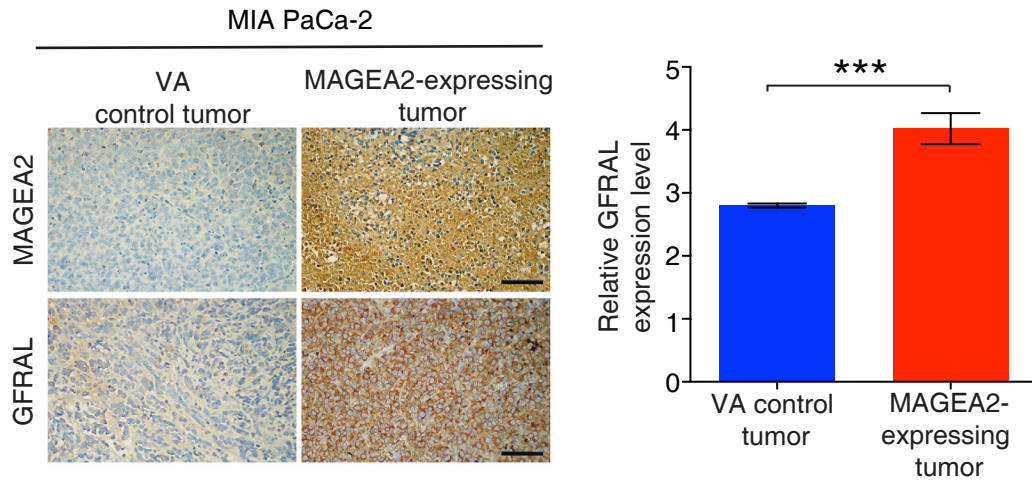

B

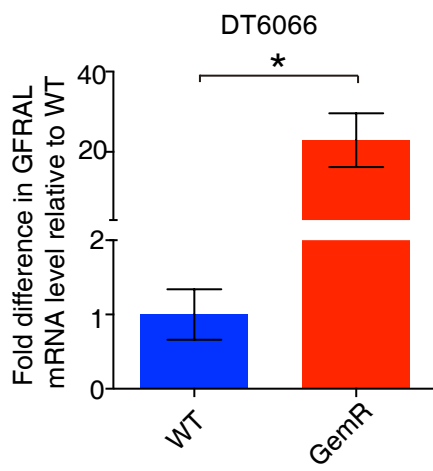

C

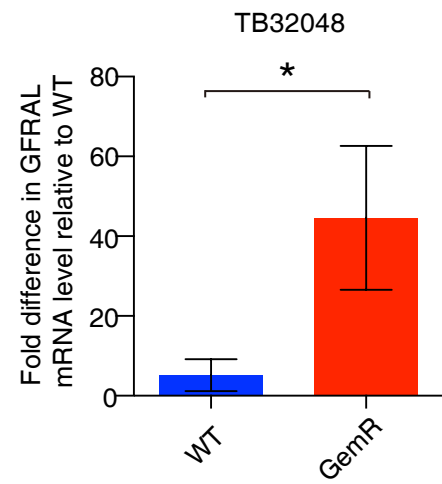

D

Parental wild type DT6066

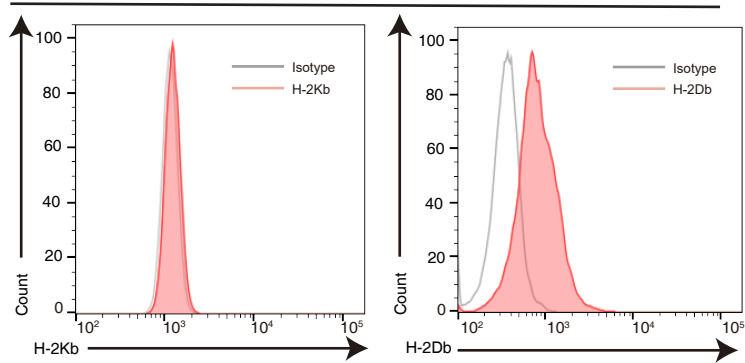

Gemcitabine resistant DT6066

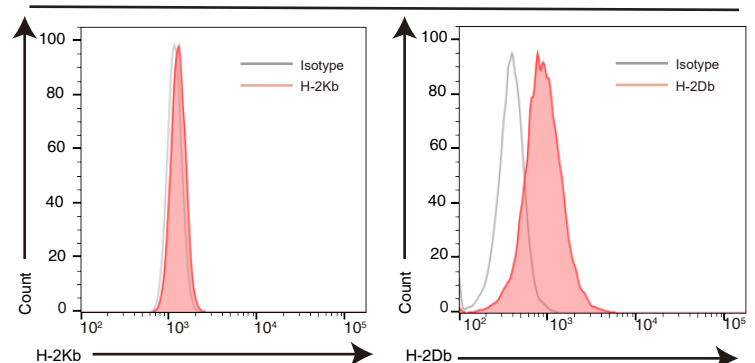

Supplement: Supplementary file 6 — Additional file 6: Fig. S4. MAGEA2 expression up-regulates the expression of GFRAL in human and murine PDAC cells both in vitro and in vivo. (A) VA- or MAGEA2-expressing tumor sections were immunohistochemically stained with MAGEA2 or GFRAL antibody. Representative immunohistochemical stained sections of MAGEA2 or GFRAL are given. Bar chart represents mean staining scores ± S.E.M. (n = 5–6 tumors per group). (B, C) RT-PCR analysis of GFRAL expression level in gemcitabine resistant (GemR) DT6066 or TB32048 cells and their parental wild type (WT) cells. Means ± S.E.M are given. (n = 3 experimental repeats). (D) FACS analysis of the expression of MHC-1 subclass on gemcitabine resistant and their parent wild type DT6066 cells respectively. *p < 0.05; ***p < 0.001. (A–C) Student’s t test. Scale bar in (A) represents 100 μm. [file 12967_2023_4519_MOESM6_ESM.pdf]

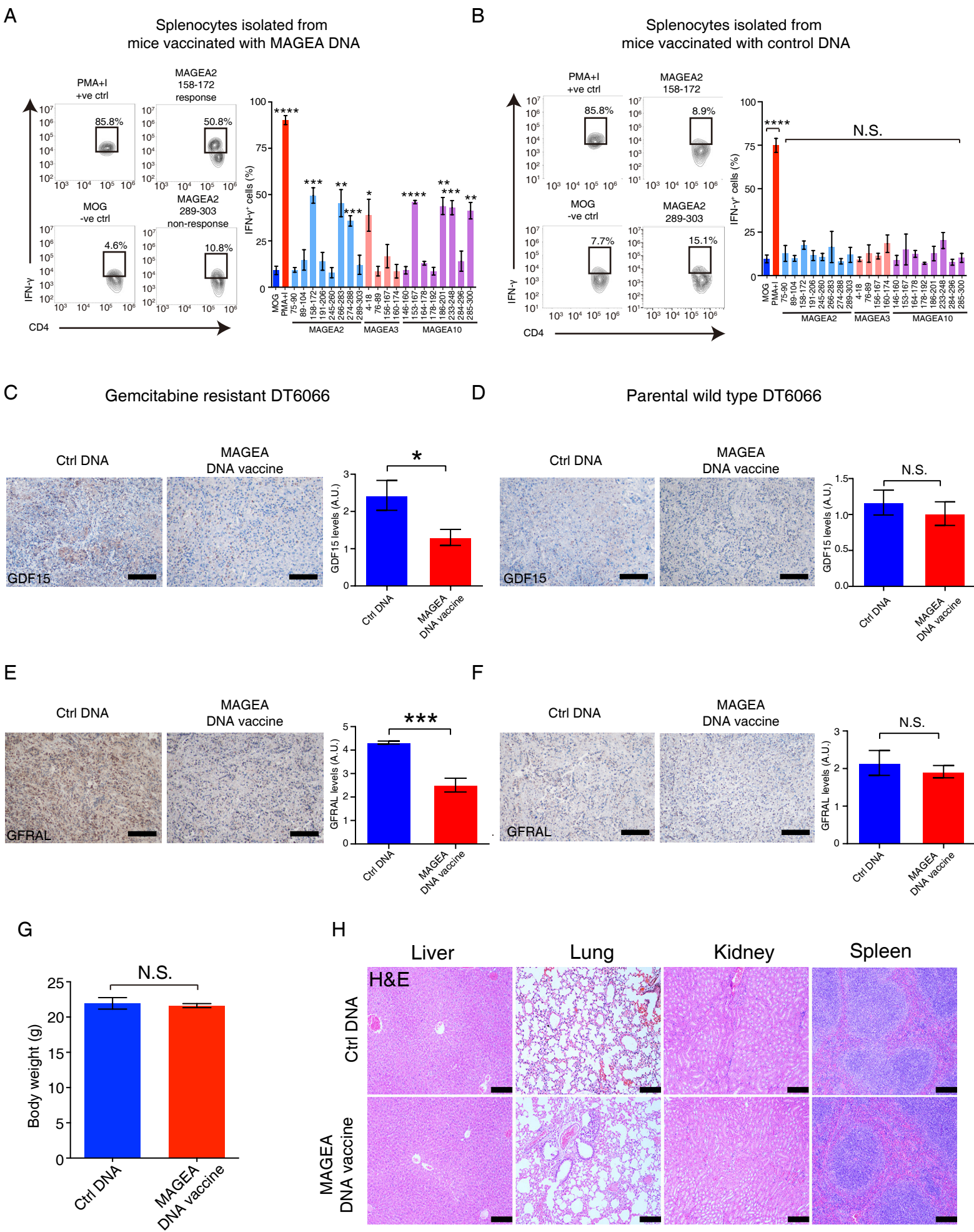

Supplement: Supplementary file 7 — Additional file 7: Fig. S5. Vaccination with multi-MAGEA antigen targeted DNA vaccine in WT C57BL/6 mice does not cause any adverse side effect. (A, B) Flow cytometry analysis of IFN- γ CD4+ T cell response to 15–16-mers peptides stimulation after vaccination with either MAGEA DNA vaccine or control DNA (n = 3 mice per group). (C–F) Representative IHC analysis of GDF15 (C, D) and GFRAL (E, F) staining on tissue sections from either GemR or WT tumors in each treatment group (n = 3 tumors analyzed per group). (G) Mouse body weights of control empty vector or MAGEA DNA vaccine treated mice. (H) Representative H&E stained sections of lung, heart, liver and spleen from the mice being immunized with either MAGEA DNA vaccine or control DNA plus poly I:C. No gross morphological defects were observed. Bar charts represent means ± S.E.M. *p < 0.05; **p < 0.01; ***p < 0.001. ****p < 0.0001. N.S. no significant difference. (A, B) One-way ANOVA. (G) Student’s t test. Scale bars in (C–F) represents 50 µm, (H) 200 µm. [file 12967_2023_4519_MOESM7_ESM.pdf]
