# Supplementary material for: Immunization with a multi-antigen targeted DNA vaccine eliminates chemoresistant pancreatic cancer by disrupting tumor-stromal cell crosstalk
Source: J Transl Med. 2023 Oct 9;21:702. doi: 10.1186/s12967-023-04519-3 (PMC10561406; doi:10.1186/s12967-023-04519-3)
Supplement: Supplementary file 8 — Additional file 8: Fig. S6. Gemcitabine or 5′FU resistant PDAC and NSCLC cell lines are cross-resistant to 5′FU and gemcitabine treatment. (A–D) RT-PCR analysis of the MAGEA expression in human 5′FU resistant PDAC cell (A, B) and NSCLC cells (C, D) respectively. (E–H) Human gemcitabine- or 5′FU-resistant PDAC cells (E, F) and NSCLC cells (G, H) were cross resistant to 5′FU and gemcitabine as compared with their parental wild type cells. (I–J) 5′FU or gemcitabine IC50 analysis of mouse gemcitabine- or 5′FU-resistant DT6066 cells and their parental wild type cells (n = 3 independent experiments). Bar charts represent means ± S.E.M. *p < 0.05; **p < 0.01; ***p < 0.001. (A–D) One-way ANOVA. [file 12967_2023_4519_MOESM8_ESM.pdf]

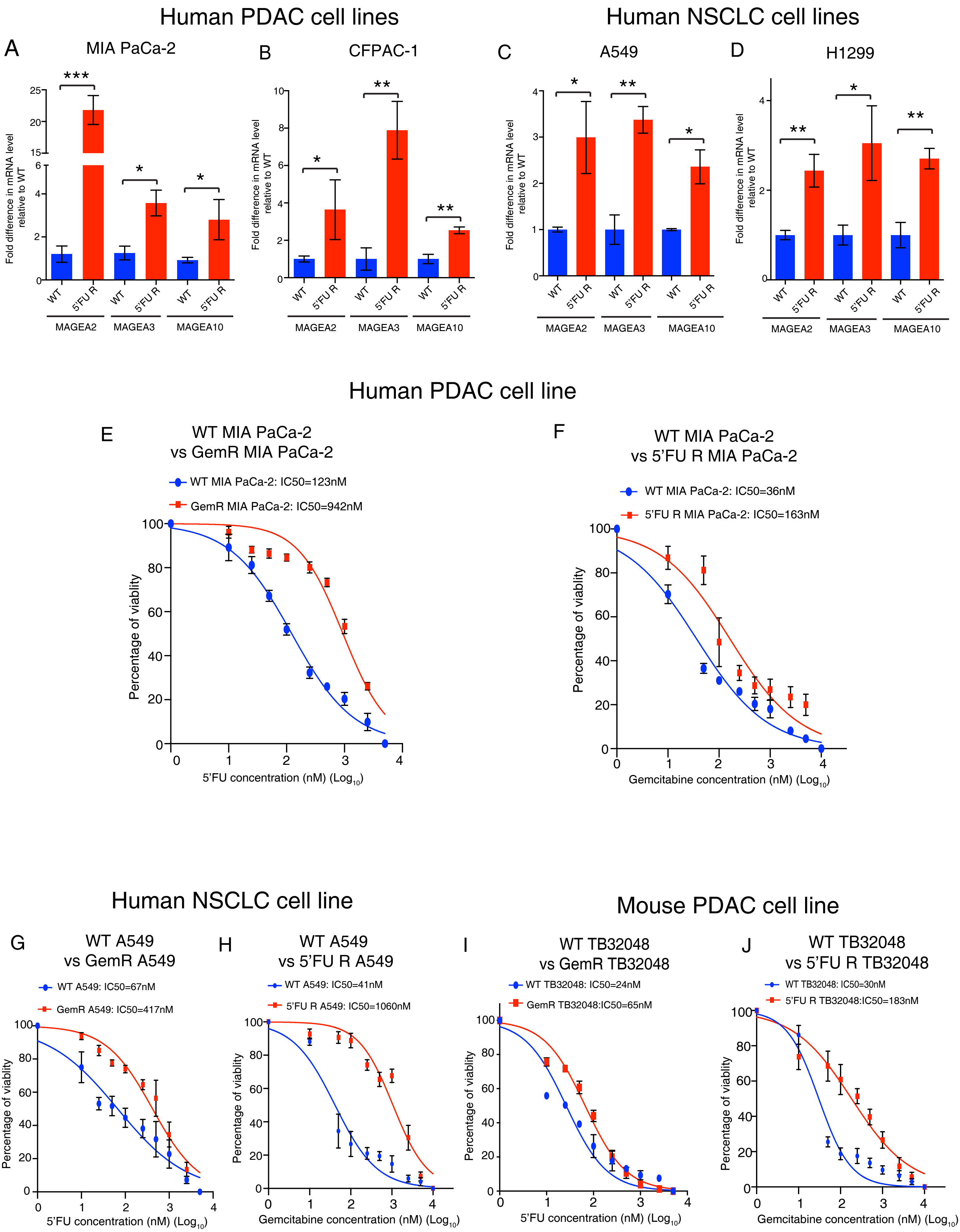
Human PDAC cell lines
Human NSCLC cell lines
A
MIA PaCa-2
***
*
*
Fold difference in mRNA level relative to WT
WT
5'FU R
MAGEA2
MAGEA3
MAGEA10
B
CFPAC-1
*
**
**
C
A549
*
**
*
D
H1299
**
*
**
Human PDAC cell line
E
WT MIA PaCa-2
vs GemR MIA PaCa-2
WT MIA PaCa-2: IC50=123nM
GemR MIA PaCa-2: IC50=942nM
Percentage of viability
5'FU concentration (nM) (Log10)
F
WT MIA PaCa-2
vs 5'FU R MIA PaCa-2
WT MIA PaCa-2: IC50=36nM
5'FU R MIA PaCa-2: IC50=163nM
Gemcitabine concentration (nM) (Log10)
Human NSCLC cell line
Mouse PDAC cell line
G
WT A549
vs GemR A549
WT A549: IC50=67nM
GemR A549: IC50=417nM
H
WT A549
vs 5'FU R A549
WT A549: IC50=41nM
5'FU R A549: IC50=1060nM
I
WT TB32048
vs GemR TB32048
WT TB32048: IC50=24nM
GemR TB32048:IC50=65nM
J
WT TB32048
vs 5'FU R TB32048
WT TB32048: IC50=30nM
5'FU R TB32048:IC50=183nM
